# SRE_BBC: A Self-Adaptive Security Enabled Requirements Engineering Approach for SLA Smart Contracts in Blockchain-Based Cloud Systems

**DOI:** 10.3390/s22103903

**Published:** 2022-05-21

**Authors:** Irish Singh, Seok-Won Lee

**Affiliations:** 1 Department of Computer Engineering, Ajou University, Suwon 16499, Korea; singhirish@ajou.ac.kr; 2 Department of Software and Computer Engineering, Ajou University, Suwon 16499, Korea; 3 Department of Artificial Intelligence, Ajou University, Suwon 16499, Korea

**Keywords:** security, attacks, vulnerabilities, goal model, threat model, self-adaptation, SLA, smart contract, blockchain, cloud, healthcare

## Abstract

Current blockchain-based cloud (BBC) systems have several security vulnerabilities regarding smart contracts (SC), and several attacks have been reported recently. The SC development lacks standard design processes that follow software lifecycle principles to model secure SC. Secondly, the security mechanisms in the SC are not constantly evolved to resist evolving adversary attacks. BBC systems lack self-adaptive security capability to make spontaneous decisions when adversarial attacks are encountered. To build a self-adaptive secure BBC system that follows standard software development lifecycle principles to model secure SC, we propose the so-called self-adaptive security RE_BBC framework. The framework would utilize the MAPE-BBC adaptation loop to make decisions internally based on the threat models, goal models, and service level agreement (SLA) SC security specifications. The framework identifies vulnerabilities and threats and takes precautionary measures using self-adaptive SC agents. We validated the proposed methodology theoretically and empirically, and statistically proved the research questions and hypothesis using the *t*-test and Mann–Whitney *U* test. Subsequently, we compare our proposed approach with the Security Quality Requirements Engineering approach (SQUARE). The feasibility results and the replicated study results indicate that the proposed approach outperformed the SQUARE approach in terms of artifacts quality, self-adaptive security evaluation quality, efficiency in response time, complexity, and usefulness of the proposed approach for the Healthcare Data Management (HDM) system. SC security developers can immensely benefit from our proposed methodology. They need not reengineer SC from scratch; depending on their security needs and plan, the contract can be adapted to execute a new plan.

## 1. Introduction

A smart contract (SC) suffers from various security vulnerabilities, risks, and trust issues with miners. The task of a miner is to assemble ordered transactions in a new block based on the incentive provided by the transactions. Malicious miners receive higher incentives from malicious users and place their transactions first in the block. Luu et al. [1] discovered that 8833 of 19,366 existing Ethereum contracts were vulnerable. For example, in June 2016, felons attacked the Smart Contract DAO [2] by intercepting a recursive call bug and stole approximately 60 million dollars. Another attack was in March 2014, where criminals misused the transaction mutability in Bitcoin to attack MtGox [3], the largest Bitcoin trading platform. As a result, MtGox cracked, and 450 million dollars of Bitcoin was stolen. These occurrences indicate that SC designers and developers lack knowledge of intrinsic security requirements, security design, and patterns. Security cannot be guaranteed because there are no effective ways to prove the correctness, reliability, and security of SC [4]. Currently, SC development procedures lack standard design processes that follow software lifecycle principles to model secure SC; subsequently, SC applications are not updated, and vulnerabilities remain unresolved [5]. Second, once employed, SC are inert to changes; hence, the security mechanisms and defense strategies need to be constantly evolved because static defense is not certain to resist evolving adversary attacks [6]. An SC needs a self-adaptive security requirements engineering (SRE) framework to identify potential security vulnerabilities and provide security specifications as countermeasure solutions to mitigate them. A blockchain-based cloud (BBC) system relies on service level agreement (SLA)-based SC, which play a critical role in ensuring trust and security [7]. The SLA SC in BBC stores the contract rules for negotiating the terms of a contract, automatically verifies the contract, and executes the agreed terms. When the payment is confirmed, the service is outsourced. The SLA SC acts as a third party among untrustworthy BBC users. SLA SC are stored in chronological order in a distributed ledger of the cloud users to enable transparency, consistency, traceability, trust, consensus, and immutability of the transactions. If any party attempts to change a contract on the BBC, other cloud users can detect and prevent the change. These characteristics of SC have also increased the complexity of SC development and caused many security vulnerabilities, such as transaction ordering dependence, timestamp dependence, mishandled exceptions, and reentrancy vulnerability. These vulnerabilities tend to be significantly costly and require substantial effort to be detected. It is critical to explore and design security challenges and countermeasures in the early phase of SC development, that is, during the SRE process of SC, self-adaptive security requirements for the current security challenges need to be provisioned in SC to detect and mitigate SC vulnerabilities and related attacks.

### Major Contributions

The objective of this study was to develop a novel self-adaptive security RE_BBC process for SLA-based SC for BBC systems to detect security vulnerabilities and challenges and mitigate them by providing proactive counter-solutions. The proposed approach also guides the process of creating a secure SC. We exploit the KAOS [8] goal modeling approach to build goal and threat models that represent an attacker’s threats, attacks, and objectives based on attack scenarios.

- We exploited our proposed RE_BBC process [7], goal model, threat model, and the MAPE-BBC adaptation process [9] to provide self-adaptive security for SLA-based SC for the BBC. The goal model provides countermeasures and answers to what countermeasures are provided, who provides these countermeasures, and how the countermeasures are provided.
- We propose the Adaptive Secure Business Contract Language (AS-BCL) and Adaptive Secure Formal Contract Language (AS-FCL) formalisms that map the concepts of formalism to the MAPE-BBC phases to provide self-adaptiveness.
- We have statistically proved the research questions and hypotheses using the *t*-test [10] and Mann–Whitney *U* test [11].
- We compared the state-of-the-art SQUARE method [12] and the proposed SRE_BBC approach based on statistical tests including mean, median, standard deviation, and *p*-values calculations on the defined parameters such as quality of artifacts and self-adaptive security evaluation quality, efficiency, complexity, and usability.
- We validated our research study with six subject matter experts who have 15+ years of experience in the software engineering field and are familiar with security concepts, Blockchain, SC, and cloud computing areas.

The validation results indicate that the proposed approach is more efficient and practical at providing self-adaptive security for SLA-based SC for BBC systems than the state-of-the-art SQUARE method.

The remainder of this paper is organized as follows. Section 2 provides an overview of the proposed approach. Section 3 describes the validation methods used to theoretically and empirically evaluate the proposed approach. Section 4 describes the Healthcare Data Management (HDM) case study adopted throughout this study to explain and evaluate our work. Section 5 discusses applying the proposed approach to a case study. In Section 6, we demonstrate the theoretical and comparative empirical results. Section 7 discusses the threats to validity. Section 8 reviews key-related studies. Section 9 addresses the current limitations, gaps, and challenges for future research. Finally, Section 10 provides concluding remarks based on our achieved results.

## 2. Proposed Methodology: MAPE-BBC Based Secure RE for SLA Smart Contract

**Figure 1:**
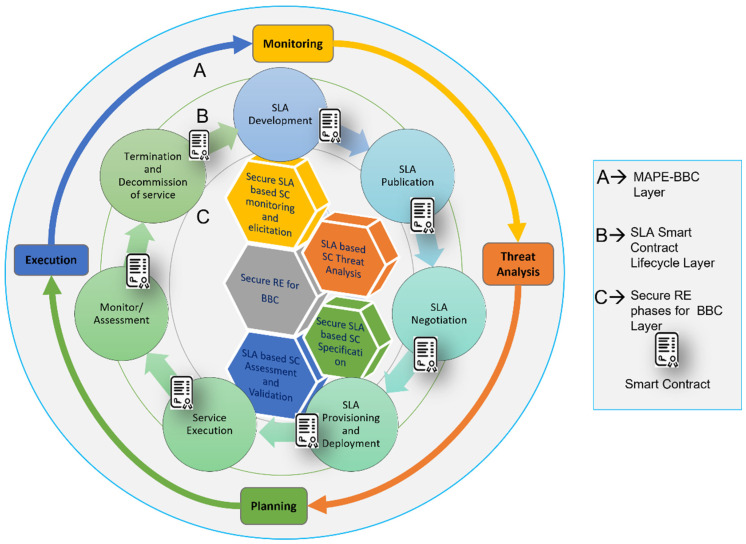
The MAPE-BBC based secure RE for SLA smart contracts.

The proposed self-adaptive security RE_BBC process aims to achieve a comprehensive understanding of the security vulnerabilities and risks in SLA-based SC for BBC systems and generate a quality set of self-adaptive security requirements for a relevant SLA-based SC that is adequate for building call integrity, atomicity, and policy compliance in BBC systems [13]. The proposed secure RE process for SLA-based SC is based on the MAPE-BBC adaptation loop, shown in Figure 1. It can be described in four phases of the SRE_BBC process according to the corresponding MAPE phases: phase 1, Secure SLA-based SC monitoring, and elicitation (monitoring). Phase 2, SLA-based SC threat analysis (threat analysis). Phase 3, Secure SLA-based SC specification for BBC (planning). Phase 4, SLA-based SC assessment and validation (execution).

### 2.1. SRE_BBC Phases for SLA-Based Smart Contract Specifications

SRE_BBC aims to design, develop, deliver, and maintain a secure software/system. Goal modeling approaches [8] in RE can be used to model the security vulnerabilities and concerns of SLA-based SC while transacting actions in the BBC, such as when developing SLA and provisioning SLA for providing Quality of Service (QoS) functionalities to users. The final security requirements of the services are the leaf goals, which are directly composed in the SLA-based SC specification document. For instance, policy compliance, call integrity, and atomicity are the final security requirements for the adaptive security of SLA-based SC [13]. After eliciting all the security requirements and completing the goal tree, a self-adaptive agent is assigned to each goal. The agents have been assigned certain responsibilities to enact when certain vulnerabilities are monitored in the block transaction log during the monitoring phase and identified during the threat analysis phase of the self-adaptive security RE_BBC process. We modeled security vulnerabilities in SLA-based SC based on certain scenarios and case studies using a threat model. The SRE_BBC process was divided into four phases.

- Phase 1: Secure SLA-based SC monitoring and elicitation (Monitoring)

In the SC monitoring and elicitation phase, the business context and security necessities of different BBC stakeholders are identified based on their initialized transactions. Then, vulnerabilities and risks are monitored in the block, which comprises the current log of transactions.

**Figure 2:**
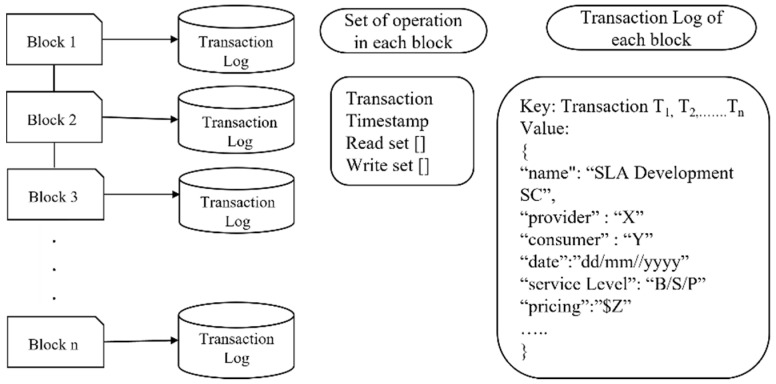
The transactions log is monitored to check vulnerabilities.

As shown in Figure 2, the current log of transactions in a block is monitored to identify the transaction ordering dependence (ToD) and/or timestamp-dependence vulnerability. Every time two transactions invoke the same SC, there is a possibility of detecting ToD, timestamp, or both. The monitor phase can clearly check the log of transactions and reveal its ToD or timestamp vulnerability using a transaction check agent.

To check the mishandled exception vulnerability, the contract call agent needs to monitor the call logs and check the return value (true/false) from the callee contract. To check whether a contract fails or executes from the output result, the contract call agent contacts the negotiator agent to check whether the negotiated SLAs are provisioned. If the negotiated SLAs are not provisioned, the contract is blocked and “Malicious Contract” is displayed. Therefore, there is a need for adaptation. In this case, we replace the non-malicious monitor/assessment contract. For the reentrancy vulnerability, the contract call agent monitors the call logs and checks the return value (true/false) from the callee contract and rollback. If the return value is false, then it is replaced with a non-aborting monitor/assessment contract.

- Phase 2: SLA-based SC Threat Analysis (Threat Analysis)

In this phase, the identified vulnerabilities, and risks in the phase 1 are analyzed to identify vulnerabilities, risks, and security violations with the help of scenarios and case studies using threat models.

For example, we defined the following scenario and case to identify ToD vulnerabilities.

**Figure 3:**
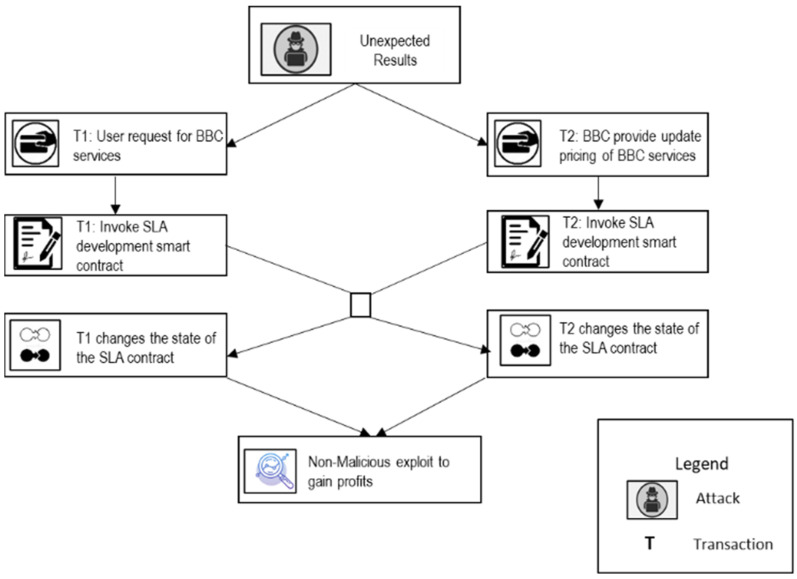
Threat Model for Case 1: WITHOUT ANY MALICE.

- Scenario A: The BBC user initializes a transaction specifying their business context and necessities, along with rewards, and broadcasts it on the BBC network. The BBC providers compete to deploy the service correctly, based on the SLA specifications and requirements of the BBC user. The first to deploy the service correctly earns a reward from the user. The user pays for the reward and consumed BBC services as per the current pricing.
- Case 1: Without Any Malice (Figure 3)

Let us assume two transactions, T_1_ and T_2_, that invoke the SLA development SC simultaneously. T_1_ is invoked by a user to request BBC services based on the specified SLA specifications and current service prices. T_2_ is invoked by the BBC provider to update the pricing rates for all services, probably to increase the prices of all current services. Because T_1_ and T_2_ invoke the SLA development contract concurrently, the next block will most likely include both transactions. The user expects to receive services based on the pricing that she/he observes when she/he submitted her/his request; however, she/he may receive a higher pricing if T_2_ is executed first. Therefore, depending on the transaction execution order, the user may need to pay much more than what she/he observed when she/he requested the services.

- Phase 3: Secure SLA-based SC Specification for BBC (Planning)

**Figure 4:**
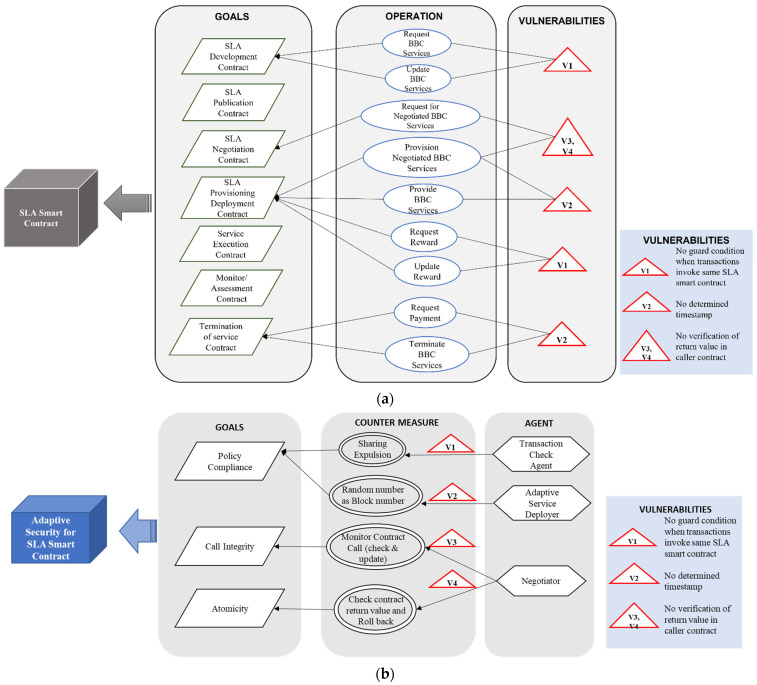
(**a**) Goal model for SLA smart contract. (**b**) Adaptive security goal model for SLA smart contract.

After the elicitation and identification of security vulnerabilities, the self-adaptive security requirements for SLA-based SC are specified in a goal tree model as shown in Figure 4. The self-adaptive security requirements (security countermeasures) are selected based on the knowledge base containing the threat models (Figure 3) and goal models of SLA-based SC (Figure 4) and provided through the SLA SC agents. The SLA-based SC specifications include self-adaptive security requirements and constraints for all vulnerabilities.

Figure 4b depicts the adaptive security goal model for SLA SC. Policy compliance, call integrity, and atomicity are considered as security goals; subsequently, four countermeasures are designed in the BBC system to accomplish these goals. Four vulnerabilities are identified in the SLA SC use cases as depicted in Figure 4a. Three agents are assigned to provide the countermeasures and take necessary actions to fulfill the goals.

Next, we define security specifications for SLA based SC which includes self-adaptive security requirements and constraints for all vulnerabilities.

- Security Specification for SLA based SC

1. Policy Compliance
  ● Sharing expulsion shall be selected as a guard condition if vulnerability V1 (ToD) is identified when requesting BBC services or updating BBC services from SLA development contract and requesting or updating a reward from SLA Provisioning & Deployment Contract.
    ◦ V_1_ indicates that a guard condition is not applied when two or more transactions invoke the same SLA SC.
    ◦ Responsible Agent: Transaction Check Agent
  ● Sharing Expulsion is a guard condition stating that, at a time, only one transaction should be invoke and execute the SC. Every transaction must request permission to execute an SC via a transaction check agent. Transaction check agents perform the entry and exit checks of the transactions and ensure that sharing expulsion is not violated.
  ● Block numbers shall be assigned as random numbers if vulnerability V2 (timestamp dependence) is identified when provisioning negotiated BBC services or providing BBC services from SLA Provisioning & Deployment Contract and requesting payment or terminating BBC services from Termination & Decommission of Service Contract.
    ◦ V_2_ indicates that there is no deterministic timestamp as attacker can easily modify it.
    ◦ Responsible Agent: Adaptive Service Deployer
2. Call Integrity
  ● The calling contract shall be monitored and updated if vulnerability V_3_ (mishandled exceptions) is identified when requesting negotiated BBC services from the SLA Negotiation Contract and provisioning negotiated BBC services from SLA Provisioning & Deployment Contract.
    ◦ V_3_ indicates that there is no verification of return value in caller contract.
    ◦ Responsible Agent: Negotiator
3. Atomicity
  ● The callee contract shall return to its previous contract, that is, the caller contract, if the callee contract fails to avoid reentrancy vulnerability (V_4_) when requesting negotiated BBC services from the SLA Negotiation Contract and provisioning negotiated BBC services from SLA Provisioning & Deployment Contract.
    ◦ V_4_ indicates that there is no verification of return value in caller contract.
    ◦ Responsible Agent: Negotiator
4. Testability Requirements
  - SLA-based SC transactions in a block shall be monitored and affirmed to assure unequivocalness, non-redundancy, precision, completeness, consistency, and trackability. These attributes shall be carried out to determine the vulnerabilities and risks of all BBC services (completeness).
5. Extrinsic Requirement
  - The BBC service transactions shall respect regulations at all times; that is, these service transactions shall be aware of data privacy and security for jurisdictional issues due to data location all the time, anywhere (legal constraints).
  - Phase 4: SLA-based SC Assessment and Validation (Execution)

In this phase, the security controls are executed and validated. For example, in Figure 4 sharing expulsion is selected and executed as a countermeasure when vulnerability V_1_ (ToD) is identified. V_1_ indicates that no guard condition is applied when two or more transactions invoke the same SLA SC. These SC security goals and countermeasures can be considered for building quality assured and secure SC for BBC applications/systems.

The validation process verifies that the resulting SLA-based SC specifications are unambiguous, non-redundant, complete, understandable, consistent, and traceable. Some problems were detected, mostly related to ambiguity and traceability. For validation, we created secure service execution scenarios and verified that the specified self-adaptive security requirements were supported. The problems were catalogued, and changes were planned. An exemplary secure service execution scenario is defined as follows:

- Self-adaptive Secure Service Execution Scenario

Dr. Lee initializes transaction T_1_ to update Mr. Kim’s EHR data and invokes the SLA healthcare provisioning and deployment contract. Mr. Kim initializes transaction T_2_ to read Mr. Kim’s EHR data and invokes the SLA healthcare provisioning and deployment contract concurrently. As T_1_and T_2_ invoke the SLA healthcare provisioning and deployment smart contract concurrently, there can be a ToD vulnerability that can violate the integrity of the EHR data. The current log of transactions in a block is monitored to identify whether a ToD or timestamp vulnerability occurs using transaction check agents and threat models. If there is a ToD vulnerability, then sharing expulsion will be executed as a guard condition. If there is a timestamp vulnerability, then as an adapted plan, block numbers are assigned as random numbers. The monitor phase alerts the system if there is a mishandled exception by monitoring the call logs and checking the return value (true/false) of the callee contract through the contract call agent. If a contract fails, the contract call agent outputs through the negotiator agent that the negotiated SLAs are not provisioned. In this case, the contract will be blocked and output as a “Malicious Contract”. In this case, the adapted plan will be executed, in which the malicious contract will be replaced with a non-malicious monitor/assessment contract. If the monitor phase identifies a reentrancy vulnerability, the contract call agent checks the return value (true/false) of the callee contract and rollback. If the return value is false, then the adapted plan is executed by replacing the aborting contract with a non-aborting monitor/assessment contract.

The proposed methodology is validated theoretically using study propositions and collected evidence. The results of the empirical study demonstrate that the proposed methodology is effective, efficient, and practical in providing self-adaptive security for BBC systems.

### 2.2. Adaptive Secure Specifications (AS-BCL and AS-FCL)

We formalized the security specifications for SLA-based SC using the proposed AS-BCL and AS-FCL frameworks.

To alleviate the lack of self-adaptive security concepts in the existing business contract language (BCL) [14] and formal contract language (FCL) [14] approaches, we proposed AS-BCL and AS-FCL as extensions of BCL and FCL, respectively. We extended the BCL and FCL language to add new concepts such as reasoning, which provides a rationale for adapting the current scenario; vulnerability, which indicates what kind of vulnerability is encountered; the current state of the SC; and responsibility, which tells who will provide the countermeasure when different vulnerabilities are encountered.

We provided mapping between the MAPE-BBC phases (monitor, threat analysis, planning, and execute phases) and AS-BCL and AS-FCL concepts to provide self-adaptiveness. However, before describing the mapping, we will explore the core concepts of AS-BCL and AS-FCL.

- Concepts of AS-BCL and AS-FCL.

Policy, role, modality, guard, reasoning, vulnerability, state, trigger, behavior, and responsibility are the core concepts of AS-BCL and AS-FCL. Next, we define each of these concepts as follows:

- **Policy** is a plan of action adopted by agents in the BBC SC to specify SC constraints.
- **Role** is the action/activity assigned to or required by stakeholders in BBC.
- **Modality** is the classification of propositions based on which one can claim necessity, possibility, or impossibility. There are three modalities: obligations, permissions, and prohibitions. These are defined as follows:
  a. **Obligation:** A Policy of an Obligation modality indicates that the event defined in the policy must occur.
  b. **Permission:** A Policy of a Permission modality indicates that the behavior defined in the policy is allowed to occur.
  c. **Prohibition:** A Policy of a Prohibition modality indicates that the behavior defined in the policy must not occur.
- **Violations**: AS-BCL and AS-FCL support expressions of guarded conditions.
- Guard is used as a predicate to determine when security countermeasures shall be enforced/will be activated [Monitor].
- **Reasoning** gives rational for adapting the current scenario [Analysis].
- **Vulnerability** states what kind of vulnerability encountered [Analysis].
- **State** is the current state of the SC.
- **Trigger** activates the policy.
- **Behavior** are countermeasures to be applied when vulnerabilities encountered [Plan].
- **Responsibility** tells who will provide the countermeasure when different vulnerabilities are encountered.
  - Example of AS-BCL for Policy Compliance Policy and its mapping to MAPE-BBC
    *Policy: PolicyCompliancePolicy*
    *Role: BBCUser/BBCProvider*
    *Modality: Obligation*
    *Guard: HasOccurred PolicyCompliancePolicyViolated [Monitor]*
    *Reasoning: NoGuardConditionWhenConcurrentTransactionsInvokeSameSLASC*
    *[Threat Analysis]*
    *Vulnerability: TransactionOrderingDependence(V1) [ Threat Analysis]*
    *State: SLADevelopmentSC*
    *Trigger: RequestBBCServices/Update BBCServices (activates the policy)*
    *Behavior: SharingExpulsion [Plan]*
    *Responsibility/Smart Contract agent: TransactionCheckAgent [Execute]*

In this example, the concepts of AS-BCL are mapped to the MAPE-BBC phases to provide self-adaptiveness. For example, the guard concept of the AS-BCL formulism is mapped to the monitor phase of the MAPE-BBC to determine when the security countermeasure shall be enforced or will be activated. Similarly, the reasoning and vulnerability concepts are mapped to the analysis phase. The reasoning concept provides a rationale for adapting the current scenario based on the vulnerability concept, which describes the type of vulnerability encountered. The behavior concept is mapped to the plan phase, which indicates what countermeasures need to be applied when different vulnerabilities are encountered. Based on the plan phase, the behavior is executed by the responsible SC agent concept value; as in this case, the transaction check agent is responsible for executing the countermeasure.

Transforming AS-BCL to AS-FCL allows us to apply the formal validation and verification procedure to a given AS-BCL program.

- Rules for AS-FCL

There are two rues for the AS-FCL based on the guard condition

***Rule 1***

if HasOccuredPolicyCompliancePolicyViolated(pid) ∉ policy:

then:

$pmap\left(pId\right)\;\leftarrow name\left(pId\right):\;trigger\left(pId\right),\;state\left(pId\right),\;reasoning\left(pId\right),\;R_{agent\left(pId\right)}behavior\left(pId\right)\;⊢\;X_{role\;}\left(pId\right)behavior\left(pId\right)$

Rule 1 denotes that, if there is no violation of the policy, the required behavior will uphold the execution of the policy

***Rule 2***

if HasOccuredPolicyCompliancePolicyViolated(pid) ∈ policy:

then:

pmap(pId) ← name(pId): trigger(pId), state(pId), reasoning(pId)

vulnerability(vId)

¬behavior(pId)

$R_{agent\left(pId\right)}behavior\left(pId\right)\;⊢\;X_{role\;}\left(pId\right)behavior\left(pId\right)$

Rule 2 is applied when there is a violation of the policy, and the required behavior does not continue.

In these two rules, Pmap(pId) is the policy ID, trigger(pId) concept activates the policy, state(pId) is the current state of the SC, reasoning(pId) provides the rationale for adapting the current scenario, vulnerability(vId) states what kind of vulnerability is encountered, and $R_{agent\left(pId\right)}$ is the agent responsible for providing the countermeasure when any vulnerability is encountered. X is a modality: X is O if Modality: Obligation, *p* if Modality: Permission, and O¬ if Modality: Prohibition. The Xrole(pId)behavior(pId) concept implies that the required behavior as per the modality is executed between the roles.

- Example of AS-FCL for Policy Compliance Policy and its mapping to MAPE-BBC

Because *‘HasOccurred pId’* Violated guard **[Monitor]** occurs in the policy, we can leverage the following mapping condition to obtain the AS-FCL rule:

if HasOccuredPolicyCompliancePolicyViolated(pid) ∈ policy:

then:

name(pId)←SR1.1

Trigger← RequestBBCServices/Update BBCServices

state(pId)← SLADevelopmentSC

reasoning(pId) ←NoGuardConditionWhenConcurrentTransactionsInvokeSameSLASC

$vulnerability\left(vId\right)\;\leftarrow \;TransactionOrderingDependence\left(V_{1}\right)$

¬behavior(pId) ←¬SharingExpulsion

$R_{agent\left(pId\right)}behavior\left(pId\right)\;\leftarrow \;R_{transactionCheckAgent\;}SharingExpulsion$

$⊢\;X_{role\;}\left(pId\right)behavior\left(pId\right)\leftarrow \;O_{BBCUser/BBCProvider}SharingExpulsion$

In this example, the concepts of the AS-FCL are mapped to the MAPE-BBC phases to provide self-adaptiveness. For example, the guard condition concept with value ‘*HasOccurred pId*’ Violated “of the AS-FCL formulism is mapped to the monitor phase of the MAPE-BBC to determine when the security countermeasure shall be enforced or will be activated. Similarly, the reasoning concept with value “NoGuardConditionWhenConcurrentTransactionsInvokeSameSLASC”, and vulnerability concept with value “$TransactionOrderingDependence\left(V_{1}\right)$” are mapped to the threat analysis phase. The reasoning concept provides a rationale for adapting the current scenario based on the vulnerability concept, which describes the type of vulnerability encountered. The $R_{agent\left(pId\right)}behavior\left(pId\right)$ concept with value “$R_{TransactionCheckAgent}SharingExpulsion$” is mapped to the Plan phase, which indicates what countermeasures need to be applied when different vulnerabilities are encountered and the responsible agent to execute the countermeasure. The Xrole(pId)behavior(pId) concept with value “$O_{BBCUser/BBCProvider}SharingExpulsion$” is mapped to the execute phase of the MAPE-BBC, which implies that the required behavior, an obligation, is executed between the BBC user and BBC provider.

## 3. Validation Method

To evaluate how the proposed approach can answer the study questions, two validation methods are conducted in this study. The first method, theoretical evaluation validates the proposed approach using study propositions and gathered evidence. However, the second method, empirical study is conducted with the subject matter experts (SMEs) to apply the SQUARE approach and the proposed approach.

### 3.1. Theoretical Evaluation

The theoretical evaluation was conducted using the case study methodology process proposed in [15] based on the case study research method [16].

#### 3.1.1. Case Study Design Methodology

The case study methodology process was conducted by applying the proposed approach to the HDM case study, which is presented in Section 4. The first step comprised clearly defining the study questions and identifying the study propositions by elaborating the study questions to answer the study questions. Accordingly, the units of analysis (measurement units) are extracted from selected resources. The analysis was performed by mapping the collected evidence to each step of the proposed methodology. The validation procedure was performed by an independent researcher who reviewed the captured evidence.

- Study Question

The following are the study questions that need to be answered to implement the self-adaptive security RE_BBC in the case study.

**SQ1**: How and why can the proposed approach address self-adaptive security design requirements for SLA-based SC in BBC systems?

**SQ2**: How can the proposed approach assess self-adaptive security achievements in the SC development framework for BBC?

- General Study Propositions

GP1: The self-adaptive security RE_BBC process can achieve its research goals because of the integrated MAPE-BBC adaptation loop, which benefits from the cooperation between the threat models, goal models, and SLA security specifications.

GP2: The self-adaptive security RE_BBC process can assess the self-adaptive security achievement by evaluating the artifacts created during the process based on specific measurement parameters.

The specific propositions that are derived from the above general propositions, are listed in Table 1.

- Units of Analysis

The self-adaptive security RE_BBC process will be applied by the SMEs to the (units) “scenarios illustrated,” “goal models,” “threat models,” “security requirements for mitigating the vulnerabilities in the SLA-based SC,” “AS-BCL,” and “AS-FCL” formalization for the HDM case study.

In addition, SMEs must be involved in the MAPE phases, namely, monitor (monitoring vulnerabilities), threat analysis (analyzing the kind of threat and its behavior), plan (planning the countermeasure), and execute (executing the countermeasure). These subjects need to be examined in a case study using this case study design.

#### 3.1.2. Data Collection Method

The self-adaptive security RE_BBC process for SLA SC is applied to the units of analysis. The generated measurement data are connected to the study propositions. The generated measurement data results will be collected by SMEs as vulnerability identification and countermeasures summary report (VICSR) sheets, presented in Table 2, and a lessons learned meeting.

From the findings via the VICSR shown in Table 2, the analysis is performed by mapping the collected evidence into each step of the proposed methodology and verifying it through the propositions we claimed. The interpretations of the findings are discussed in theoretical result subsection of Section 6.

### 3.2. Empirical Study

The main objective of the empirical study was to determine the usefulness of the proposed methodology in terms of performance parameters, such as efficiency (response time), effectiveness in identifying vulnerabilities, and providing countermeasures, and to determine the complexity level for building self-adaptive security for SLA-based SC for BBC systems.

- Study Question and Hypothesis

The following study questions and hypotheses are defined to analyze and compare the proposed methodology, self-adaptive security RE_BBC with security quality requirements engineering (SQUARE) [12]. The study questions and their respective hypotheses and parameters are presented in Table 3.

**SQ3**: How efficaciously does the proposed approach identify the security goals and SC vulnerabilities, gather security requirements, and provide countermeasures to mitigate the vulnerabilities via SC agents?

**SQ4**: How efficient is the proposed approach for addressing self-adaptive security concerns in SC development for BBC systems?

**SQ5**: Can the proposed approach be considered realistic for providing self-adaptive security for BBC systems?

#### 3.2.1. Feasibility Study

The feasibility study evaluates how effectively self-adaptive security RE_BBC can address self-adaptive security vulnerabilities and provide security countermeasures in HDM applications. This study asserts that self-adaptive security RE_BBC can efficiently identify more vulnerabilities and more security requirements and provide complete countermeasures to mitigate those vulnerabilities in a time-efficient manner compared with the SQUARE process. The empirical study is conducted as a controlled experiment [17] with SMEs from an industrial and academic backgrounds with more than 15 years of experience.

- Participants

The SMEs were selected through a close recruitment process based on the following criteria:

◦ The SMEs should have at least 10 years of experience in software engineering field.
◦ The SMEs should be familiar with SRE process and security concepts.
◦ The SMEs should have knowledge about Blockchain, SC and cloud computing.

Six SMEs were selected from renowned multinational industries and academic institutions for the feasibility study.

- Study Design

The SMEs were divided into two groups (groups 1 and 2), with three SMEs in each group. Group 1 applied the SQUARE approach [12], whereas group 2 performed the proposed approach self-adaptive security RE_BBC.

The feasibility study was conducted in two sessions. In the first session, the group 1 SMEs were asked to perform the SQUARE methodology [12]. The following steps were followed to conduct session one:

◦ Domain application introduction: A description of the HDM is provided to SMEs.
◦ Step 1: Agree on definitions: The first task for SMEs is to agree upon a common set of general security definitions and HDM security goals. The agreement results and time required to complete this step are recorded.
◦ Step 2: Identify security goals: SMEs are asked to identify the security goals of SLA-based SC. The security goals for the SLA-based SC and time required to complete this step are recorded.
◦ Step 3: Select elicitation techniques: SMEs choose from various elicitation techniques. The selected elicitation technique and time required to complete this step are recorded. (Goal and threat models)
◦ Step 4: Develop artifacts to support the elicitation technique: SMEs are asked to develop artifacts such as attack scenarios, network maps, diagrams, attack tree diagrams, and use and misuse cases. Based on the developed artifacts, SMEs are asked to identify SC risks and vulnerabilities, invoked SLA SC, types of vulnerabilities, and use cases in which these vulnerabilities are encountered. The SC vulnerabilities, invoked SLA SC, type of vulnerability, use cases, and time required to complete this step are recorded.
◦ Step 5: Elicit security requirements: The artifacts developed in step 4 are used to develop the initial requirements. The initial requirements and time required to complete this step are recorded.
◦ Step 6: Categorize requirements based on levels and constraints: The requirements elicited in step 5 are subsequently categorized to meet the needs of the HDM security goals. The categorized requirements and time required to complete this step are recorded.
◦ Step 7: Perform risk assessment: Risk assessment allows the discovery of how the combination of the impact and likelihood of various threats identified affects HDM risk tolerance with regard to each categorized requirement. SMEs are asked to plan security requirements to mitigate the identified risks and vulnerabilities and identify the software agents responsible for providing these security requirements. The security countermeasure solutions (security requirements), software agents, and time required to complete this step are recorded.
◦ Step 8: Prioritize the requirements: Based on the categorized requirements identified in step 6 and risks and vulnerabilities identified in step 7, prioritize the requirements. The prioritized requirements and time required to complete this step are recorded.
◦ Step 9: Requirement inspection: The final list of prioritized requirements is inspected by the SMEs from group 2. The inspection results and time required to complete this task are recorded.
◦ The final output of SQUARE is a security requirements document designed to satisfy the security goals of the HDM BBC case study.
◦ Formal security specifications provision: A set of AS-BCL and AS-FCL formal security specifications are provided to SMEs.
◦ Formal security specification evaluation: SMEs are asked to evaluate the formal security specifications based on their defined security requirements and artifacts that evaluate the self-adaptive security concepts by mapping the AS-BCL and AS-FCL formalization concepts (guard, reasoning, vulnerability, behavior, and responsible agent) based on the MAPE-BBC phases and verify their values. The captured security requirement achievements and time required to complete this step are recorded.
◦ Study survey: At the end of the session, a survey questionnaire was conducted to collect feedback from SMEs about the experiments.

In the second session, the proposed approach “Self-adaptive security RE_BBC” is presented to the SMEs from group 2, who were asked to collect the underlined italic artifacts in each phase of the proposed methodology from Table 2: “Evidence Collection through VICSR”. The following steps were followed throughout the session:

◦ Domain application introduction: A description of HDM is provided to SMEs.
◦ The proposed approach “Self-adaptive security RE_BBC” is presented to SMEs who were trained to use the proposed approach. SMEs are provided with Table 2, which guides them about the artifacts that need to be collected in each phase and from where these artifacts need to be collected.
◦ Phase 1: Security requirements elicitation and SC monitoring with “Self-adaptive security RE_BBC”: SMEs are asked to identify the security goals, stakeholders, and security requirements for SLA-based SC. The security goals, stakeholders, and security requirements for SLA-based SC and time required to complete this phase are recorded.
◦ Phase 2: Threat analysis with the proposed approach: SMEs are asked to identify SC vulnerabilities, invoked SLA SC, types of vulnerabilities, and use cases in which these vulnerabilities are encountered. The SC vulnerabilities, invoked SLA SC, type of vulnerability, use cases, and time required to complete this phase are recorded in this step.
◦ Phase 3: SLA specification using the proposed approach: SMEs are asked to plan security countermeasure solutions or security specifications to mitigate the vulnerabilities identified in phase 2 and identify the software agents responsible for providing these security specifications. The security countermeasure solutions (security specifications), software agent, and time duration to complete this phase are recorded.
◦ Phase 4: SLA-based SC assessment and validation using the proposed approach: The SMEs of group 1 check that the resulting SLA-based SC specifications are persistent, correct, absolute, asserted, and valid by monitoring and evaluating the specified self-adaptive security requirements using secure service execution scenarios.
◦ Formal security specification provision: A set of AS-BCL and AS-FCL formal security specifications are provided to SMEs.
◦ Formal security specification evaluation: SMEs are guided to use an assessment method to evaluate the formal security specification based on their defined security requirements. The captured security requirement achievements and time required to complete this step are recorded.
◦ Study survey: At the end of the session, a survey questionnaire was conducted to collect feedback from SMEs about the experiments.

#### 3.2.2. Replicated Study

The replicated study addresses the generalization of new vulnerabilities that were not detected during the first inspection of the feasibility study. This study aimed to evaluate the practicality of “self-adaptive security RE_BBC”. The replicated study was also conducted as a controlled experiment designed [17] with SMEs from various industrial and academic backgrounds with more than 15 years of experience.

- Participants
  ◦ The participants in the replicated study were selected based on the same criteria as in the feasibility study.
  ◦ Six SMEs were selected from renowned multinational industries and academic institutions for the replicated study.
- Study Design

The SMEs were divided into two groups (groups 1 and 2), with three SMEs in each group. Group 1 applied the proposed approach “Self-adaptive security RE_BBC,” whereas group 2 applied the SQUARE methodology [12].

The replicated study was conducted in two sessions. During the first session, the proposed approach “Self-adaptive security RE_BBC” was presented to the SMEs of group 1 who were asked to collect the underlined italic artifacts in each phase of the proposed methodology from Table 2: “Evidence Collection through vulnerability identification, and Countermeasures Summary Report”. The feasibility study, session two steps were followed to conduct session one of the replicated study During the second session, the group 2 SMEs were asked to perform the security quality requirements engineering (SQUARE) methodology [12] based on the same steps as in session 1 in the feasibility study, except step 9: requirements inspection, where the final list of the prioritized requirements were inspected by the SMEs of group 1 of the replicated study. 

## 4. Case Study: Healthcare Data Management-Blockchain-Based Cloud

**Figure 5:**
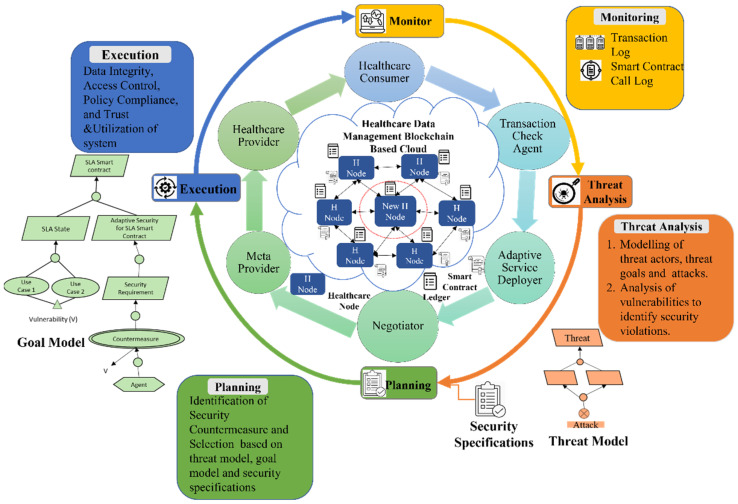
MAPE-BBC adaptation loop for SLA healthcare smart contract in BBC systems.

The healthcare data management-BBC (HDM-BBC), shown in Figure 5, is an emerging technology that can safely store healthcare data records on a cloud. HDM-BBC [18] ensures that the healthcare records transparent and open to healthcare users, improving the reliability, performance, and quick and secure service provisioning through the healthcare data management-SC (HDM-SC) in the HDM-BBC. The stakeholders of the HDM-BBC are patients (new and old patients), doctors, pharmacies, and insurance companies. The SC is signed among all stakeholders. The HDM-BBC provides patient data-as-a-service (PDaaS), doctor-as-a-service (DaaS), insurance-as-a-service (INaaS), and pharmacy-as-a-service (PHaaS). Through PDaaS, patients can access their data anywhere and anytime, and only authorized doctors can access and update the patient records. Through DaaS, any patient can consult a doctor based on their requirements and needs. INaaS provides insurance claims to patients based on patients’ healthcare expenses and insurance. The PHaaS provides medicine based on doctors’ prescriptions.

### SLA-Based Healthcare Data Management-Blockchain-Based Cloud (HDM-BBC) Smart Contracts

SLA-based HDM-BBC SC are mutual agreements between two or more HDM-BBC stakeholders who participate in HDM-BBC service exchanges through transactions. They specify security requirements and specifications such as data integrity, access control (authorization), and policy compliance (data protection) for the users and stakeholders involved in the contact. If the specified service level agreements and QoS are not met, then certain penalties are imposed on stakeholders. SLA-based HDM-BBC contracts often contain security vulnerabilities and risks (e.g., ToD, timestamp dependency, and mishandled exceptions), which must be monitored, identified, and mitigated. To address these security vulnerabilities, the MAPE-BBC adaptation loop is exploited to monitor, identify, and mitigate security vulnerabilities via security countermeasure solutions or security requirements.

Accordingly, there are some mandatory functional and security requirements, as follows:

- Functional Requirements

FR1: The patient must be able to access his/her data.

FR2: The authorized doctor must be able to access and update the patient records.

FR3: The authorized pharmacy must be able to access the patient healthcare prescription.

FR4: The insurance team must be able to access patient data and provide health insurance claims to patients.

- Security Requirements

SR1: To preserve the integrity of patient data, the security requirement asserts that no two transactions shall concurrently invoke an SLA healthcare SC. This is also termed as “sharing expulsion”.

SR2: To prevent unauthorized access to the EHR, the security requirement states that only authorized users shall be permitted to access or update the EHR data.

SR3: To prevent malicious attacks from sharing the EHR data located in the HDM-BBC from one country to another, it is required that EHR data comply with the standard data security rules and regulations of the country where the data are being shared. For example, if healthcare data need to be sent from South Korea to the United States (US), then the data must comply with the Health Insurance Portability and Accountability Act (HIPAA) standard of the US [19].

- Scenarios

**Scenario 1 [Loss of Data Integrity]:** Transaction orders can change the security control that must be provisioned. For example, if one transaction invokes a healthcare service provisioning and deployment SC; then, other transactions shall wait until the first transaction exists from the SC to satisfy the security requirement SR1. Similarly, if an authorized doctor requests service provisioning and deployment of an SC to update patient healthcare data, authorization to access the patient data should be revoked by the patient, pharmacist, and insurance team until the doctor has an SC to preserve the integrity of patient healthcare data.

**Scenario 2 [Loss of Access Control]:** An authorized transaction timestamp can change the security control that needs to be provisioned. For example, no two unauthorized transactions shall be invoked in a row by changing the block timestamp to satisfy security requirement SR2. Similarly, if an unauthorized user or a potentially malicious user tries to access the EHR data and consequently update the EHR data, the access control mechanism must be adapted to avoid the user from accessing or updating the data. For example, if an unauthorized doctor requests the SLA Healthcare Development Contract to access or update the patient EHR data, then permission to access or update the patient data should be revoked by the unauthorized doctor until the patient himself/herself authorizes the doctor to access or update his/her EHR data to guarantee access control.

**Scenario 3 [Policy Non-Compliance]:** Non-monitoring of calling contracts in SLA HDM-SC can change the security control that needs to be provisioned. For example, when SLA HDM-SC A calls another SLA HDM-SC B, and if B runs abnormally, it will stop running and return false. At this point, contract A must explicitly check the return value from B to check the call execution to satisfy security requirement SR3. Similarly, if a patient requests the Healthcare SLA Negotiation Contract to share his/her data with another doctor in another country, then the EHR data will be provisioned by the Healthcare SLA Provisioning & Deployment contract (A) following the standard security rules and regulations for data protection of the country in which the data are being shared. The provisioned data from the healthcare SLA provisioning and deployment contract are then monitored by the healthcare SLA monitor/assessment contract (B) to check whether the data comply with the data protection policies of another country. If, at this point, B fails, then A shall check the return value of contract B and identify whether the request has been provisioned.

## 5. Proposed Methodology Applied to the Healthcare Data Management Blockchain Based Cloud

### 5.1. Monitoring

**Figure 6:**
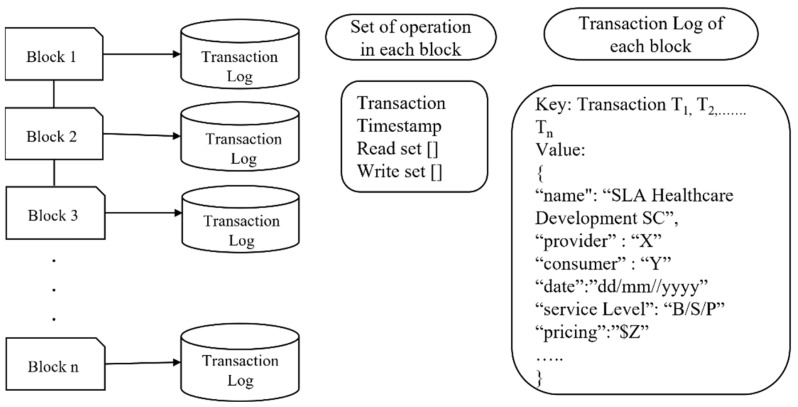
The transactions log is monitored to check vulnerabilities.

The current log of transactions in a block is monitored to identify vulnerabilities, as shown in Figure 6 below. Every time two transactions invoke the same SC, there is the possibility of ToD, timestamp, or both. The monitor phase can clearly check the log of transactions and reveal its ToD or timestamp vulnerability using a transaction check agent.

To check the mishandled, the contract call agent needs to monitor the call logs and check the return value (true/false) from the callee contract. To check whether a contract fails or executes from the output result, the contract call agent will contact the negotiator agent to check whether the negotiated SLAs are provisioned. If the negotiated SLAs are not provisioned, block the contract and display a malicious contract. Therefore, there is a need for adaptation. In this case, we replace the non-malicious Monitor/Assessment contract. For reentrancy, the contract call agent monitors the call logs and checks the return value (true/false) from the callee contract and rollback. If the return value is false, then replace it with a non-aborting monitor/assessment contract.

### 5.2. Threat Analysis

The vulnerabilities and threats in SC are analyzed to identify security violations. Four types of SC vulnerabilities (V) are described using attack scenarios and threat models [10] for the HDM domain.

#### 5.2.1. Transaction Ordering Dependence (V_1_)

When a new block contains two transactions, T_i_ and T_j_, which invoke the same SLA healthcare SC, it may trigger a transaction order dependence vulnerability

**Figure 7:**
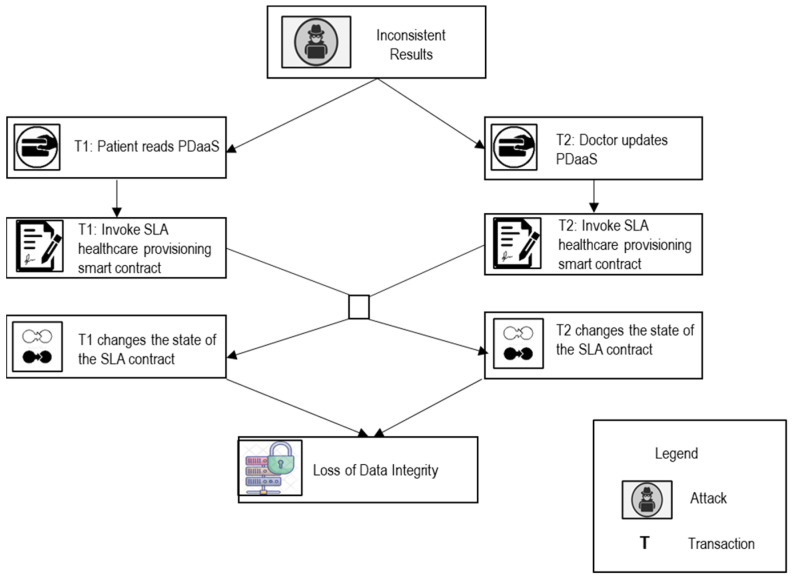
Threat Model for transaction ordering dependence scenario 1: Loss of data integrity.

- Scenario 1: Loss of Data Integrity (Figure 7)

Let us assume two transactions T_1_ and T_2_ invoke the SLA healthcare provisioning and deployment contract concurrently. T_2_ is invoked by a patient to read their EHR data using PDaaS. T_1_ is invoked by a doctor to update or write the EHR data of the patient. Because T_1_ and T_2_ invoke the SLA healthcare provisioning and deployment SC concurrently, the next block will most likely include both transactions. The patient expects to receive updated and consistent health records when (s)/he submitted her/his request, but (s)/he may receive inconsistent health records if T_2_ is executed first. Therefore, depending on the transaction execution order, the patient may receive inconsistent health records when (s)/he or she requested services.

#### 5.2.2. Timestamp Dependence (V_2_)

In an SLA-based SC in the blockchain, every state in the block has a timestamp. The trigger conditions of some SLA healthcare SC depend on the timestamp, which is set by the miner according to its local system time. If an attacker can modify it, the timestamp-dependent contracts are vulnerable.

**Figure 8:**
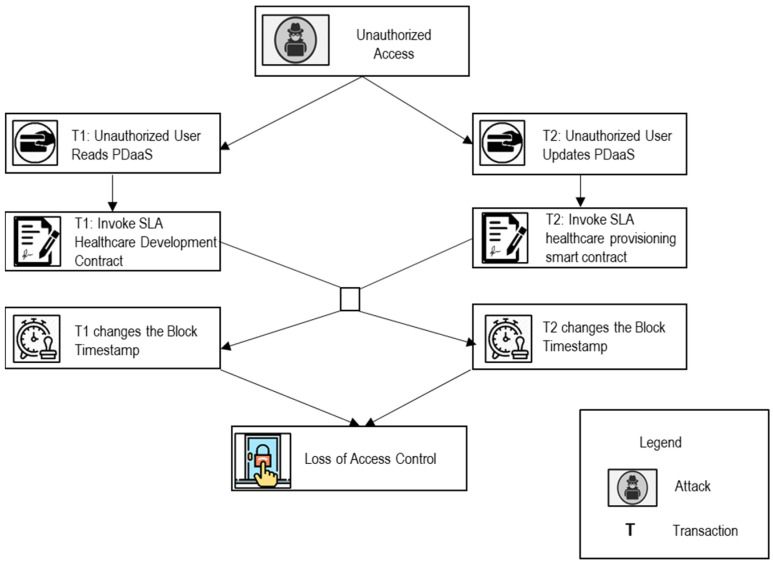
Threat model for timestamp dependence scenario 2: Loss of access control.

- Scenario 2: Loss of Access Control (Figure 8)

An unauthorized user gives the miner more incentive to invoke two transactions in a row by changing the block timestamps. The first transaction reads PDaaS, and the second transaction updates it. The malicious miner gives access to an unauthorized user to perform two transactions on the patient’s data, causing a loss of access control.

#### 5.2.3. Mishandled Exceptions (V_3_)

This category of vulnerability may occur when different SLA healthcare SC are called. When SLA healthcare contract A calls SLA healthcare contract B, if B runs abnormally, it will stop running and return false. In some invocations, contract A must explicitly check the return value to verify whether the call has been executed properly. If A does not correctly check exception information, it may be vulnerable.

**Figure 9:**
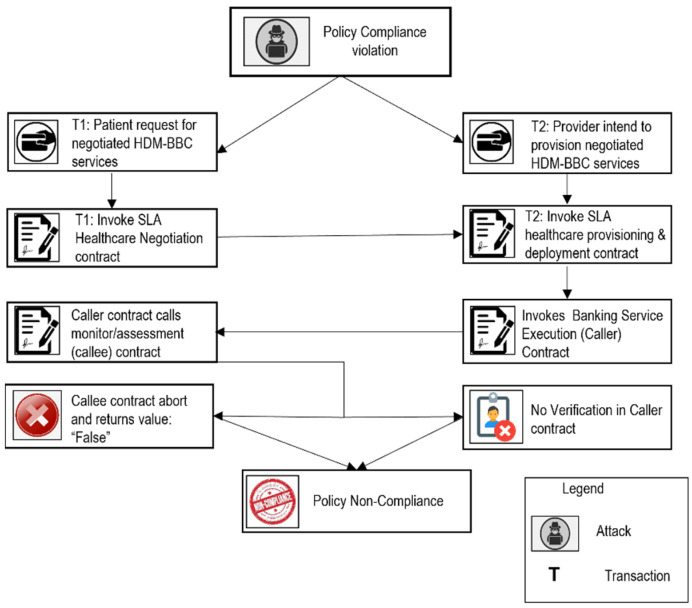
Threat model for mishandled exceptions scenario 3: Policy non-compliance.

- Scenario 3: Policy Non-Compliance (Figure 9)

The patient requests negotiated services and invokes an SLA healthcare negotiation contract. The provider intends to provide a negotiated request from the patient and invoke an SLA healthcare provisioning and deployment contract. The SLA healthcare provisioning and deployment contract invokes the healthcare service execution contract, in turn, the healthcare service execution (caller) contract calls the healthcare monitor/assessment (callee) contract. A malicious callee contract always runs abnormally and returns a true result. In this situation, it will always be considered that negotiated healthcare SLAs are provisioned, and the reward will always be paid to the healthcare BBC provider.

#### 5.2.4. Reentrancy Vulnerability (V_4_)

During the invocation of an SC, the actual state of the contract account changes after the call is completed. An attacker can use the intermediate state to make repeated calls to an SC. If an invoked contract involves a billing transaction, it may result in illegal money stealing. The SC contains a set of rules through which the SC parties agree to interact with each other.

**Figure 10:**
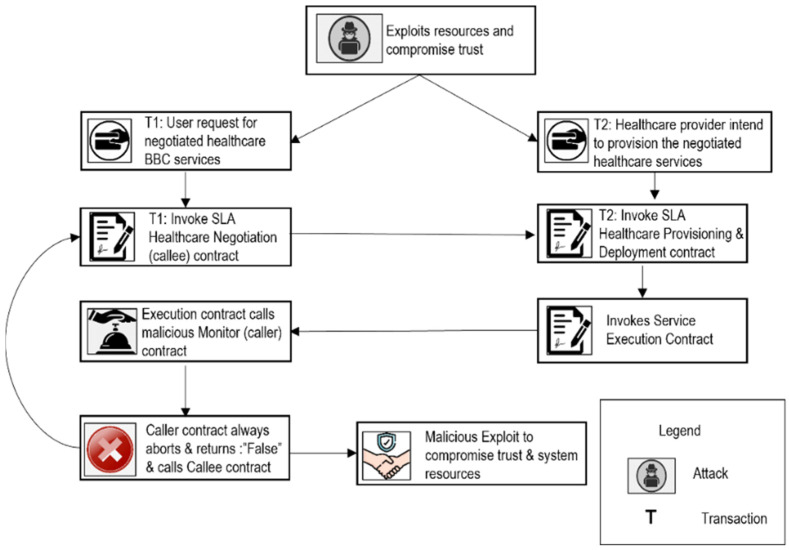
Threat model for reentrancy vulnerability scenario 4: compromised trust and system resources.

- Scenario 4: Compromised Trust and System Resources (Figure 10)

A malicious healthcare monitor/assessment (caller) contract maliciously returns that the negotiated healthcare SLAs are not provided and invokes the SLA healthcare negotiation (callee) contract. The callee again requests and responds with the provisioned services via SLA healthcare provisioning and deployment contract. The SLA healthcare provisioning and deployment contract, in turn, invokes a healthcare service execution contract for provisioned service execution. After a service is executed, it invokes a malicious caller contract for service monitoring. This loop continues to rotate endlessly and negotiated healthcare SLAs are never provided. The malicious caller contract holds up the processing of the system, and as a result, the time and cost are maximized. Second, the reputation of the provider is ruined, and trust is compromised.

### 5.3. Planning

In the planning phase, security countermeasures are selected based on the threat model, goal model, and security specifications for HDM.

**Figure 11:**
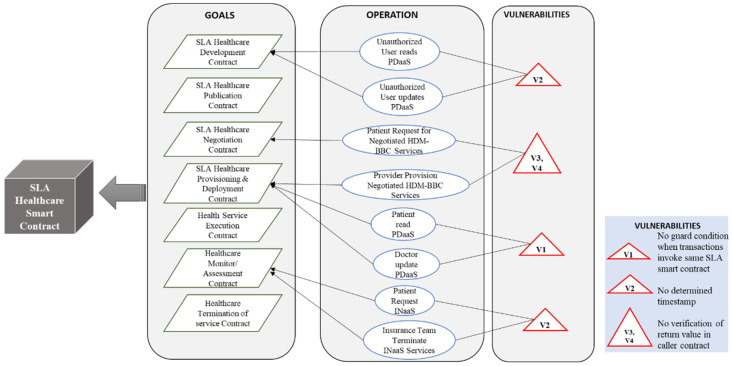

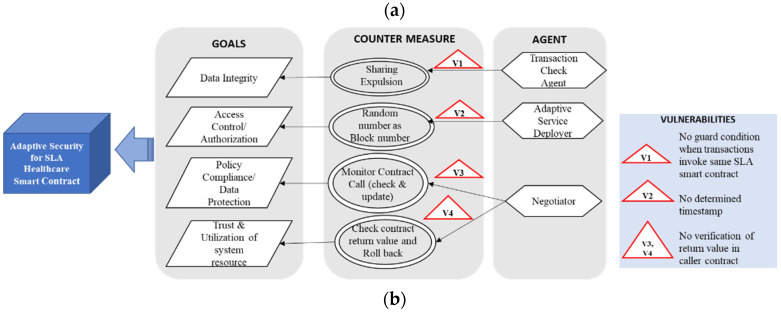
(**a**) Goal model for SLA healthcare smart contract. (**b**) Adaptive security goal model for SLA healthcare smart contract.

Figure 11b depicts the adaptive security goal model of the SLA healthcare SC. Data integrity, access control, policy compliance, and trust and utilization of system resources are considered security goals. Four countermeasures are designed in the HDM BBC system to accomplish these goals. Four vulnerabilities are identified in SLA healthcare SC use cases, as depicted in Figure 11a. Three agents are assigned to provide the countermeasures and security requirements necessary to fulfill these goals.

### 5.4. Execution

Security controls are executed in this phase. For example: Sharing Expulsion as a guard condition and countermeasure shall be selected when vulnerability V_1_ (ToD) is identified when the SLA healthcare provision and deployment contract is invoked by two transactions concurrently. In the first transaction, the patient reads the patient data using the PDaaS service; in the second transaction, the doctor updates the patient data.

V_1_ indicates that a guard condition is not applied when two or more transactions invoke the same SLA SC. The agent responsible for executing the countermeasure is the transaction check agent.

Sharing Expulsion (countermeasure) is a guard condition that states that, at a time, only one transaction should be invoked, and the SC should be executed. Every transaction must request permission to execute an SC via a transaction check agent. The transaction check agent maintains the transactions’ entry and exit check and ensures that Sharing Expulsion shall not violate.

### 5.5. Formal Representation of SLA Based Healthcare-Blockchain Based Cloud Smart Contracts Using AS-BCL and AS-FCL

Following are the formal specifications of SLA based Healthcare-Blockchain Based Cloud Smart contracts using AS-BCL and AS-FCL for the above three scenarios

- AS-BCL For Scenario 1

*Policy: DataIntegrityPolicy*

*Role: Patient/Doctor*

*Modality: Obligation*

*Guard: HasOccurredDataIntegrityPolicyViolated*

*Reasoning: NoGuardConditionWhenConcurrentTransactionsInvokeSameSLA-HDM-SC*

*Vulnerability: TransactionOrderingDependence(V_1_)*

*State: SLAHealthcareProvisioning&DeploymentSC*

*Trigger: PatientReadPDaaS/DoctorupdatePDaaS* (activates the policy)

*Behavior: SharingExpulsion*

*Responsibility: TransactionCheckAgent*

- AS-FCL for Scenario 1

Because ‘*HasOccurred pId’* Violated guard occurs in the policy, we can use the following condition of the mapping to obtain the FCL rule:

if HasOccuredPolicyCompliancePolicyViolated(pid) ∈ policy:

then:

name(pId)←SR1

trigger←PatientReadPDaaS/DoctorupdatePDaaS

state(pId)← SLAHealthcareProvisioning&DeploymentSC

reasoning(pId) ←NoGuardConditionWhenConcurrentTransactionsInvokeSameSLA−HDM−SC

$vulnerability\left(vId\right)\;\leftarrow \;TransactionOrderingDependence\left(V_{1}\right)$

¬behavior(pId) ←¬SharingExpulsion

$R_{agent\left(pId\right)}behavior\left(pId\right)\;\leftarrow \;R_{transactionCheckAgent\;}SharingExpulsion$

$⊢\;X_{role\;}\left(pId\right)behavior\left(pId\right)\leftarrow \;O_{Patient/Doctor}SharingExpulsion$

- Similar cases are:
  ◦ When the patient reads the INaaS and concurrently insurance team updates the INaaS.
  ◦ When the insurance team reads the PDaaS (to check patient details, healthcare expenses, and patient healthcare insurance) to provide insurance claims to patients, and concurrently, the doctor/pharmacist updates the PDaaS.
  ◦ When the pharmacist reads PDaaS (to check the patients’ deatals, doctors’ prescription) to provide medicines to the patient, and concurrently, the doctor updates the PDaaS.
  ◦ When patient reads the PHaaS and concurrently pharmacist updates the PHaaS.
- AS-BCL for Scenario 2

*Policy: AccessControlPolicy*

*Role: UnauthorizedUser*

*Modality: Permission*

*Guard: HasOccurred AccessControlPolicyViolated*

*Reasoning: NoDeterministicTimestamp*

*Vulnerability: TimestampDependence(V_2_)*

*State: SLAHealthcareDevelopmentSC*

*Trigger: UnauthorizedUserReadsPDaaS/UnauthorizedUserUpdatesPDaaS*

*Behavior: SetRandomNumbersasBlockNumbers*

*Responsibly: AdaptiveServiceDeployerAgent*

- AS-FCL for Scenario 2

if HasOccuredPolicyCompliancePolicyViolated(pid) ∈ policy:

then:

name(pId)←SR2

trigger← UnauthorizedUserReadsPDaaS/UnauthorizedUserUpdatesPDaaS

state(pId)← SLAHealthcareDevelopmentSC

reasoning(pId) ← NoDeterministicTimestamp

$vulnerability\left(vId\right)\;\leftarrow \;TimestampDependence\left(V_{2}\right)$

¬behavior(pId) ←¬SetRandomNumbersasBlockNumbers

$R_{agent\left(pId\right)}behavior\left(pId\right)\;\leftarrow \;R_{AdaptiveServiceDeployerAgent}SetRandomNumbersasBlockNumbers$

$⊢\;X_{role\;}\left(pId\right)behavior\left(pId\right)\leftarrow \;P_{UnauthorizedUser}SetRandomNumbersasBlockNumbers$

- AS-BCL for Scenario 3

*Policy: PolicyCompliancePolicy*

*Role: HDM-BBCUser/MBBCProvider*

*Modality: Obligation*

*Guard: HasOccurred PolicyCompliancePolicyViolated*

*Reasoning: NegotaitedSLAsNotProvisioned*

*Vulnerability: MishandledExceptions(V_3_)*

*State: SLAHealthcareNegotiationSC*

*Trigger: RequestNegotiatedHDM-BBCServices/ProvisionNegotiatedHDM-BBCServices*

*Behavior: BlockMaliciousContract&ReplaceWithNonMaliciousMonitor&AssessmentContract*

*Responsibility: NegotiatorAgent*

- AS-FCL for Scenario 3

if HasOccuredPolicyCompliancePolicyViolated(pid) ∈ policy:

then:

name(pId)←SR3

trigger← RequestNegotiatedHDM_BBCServices

/ProvisionNegotiationHDM_BBCServices

state(pId)← SLANegotiationSC

reasoning(pId) ← NegotaitedSLAsNotProvisioned

vulnerability(vId) ← MishandledExceptions(V3)

¬behavior(pId)←¬BlockMaliciousContract&

¬ReplaceWithNonMaliciousMonitor&AssessmentContract

$R_{agent\left(pId\right)}behavior\left(pId\right)\;\leftarrow R_{Negotiator\;Agent}BlockMaliciousContract\&$

ReplaceWithNonMaliciousMonitor&AssessmentContract

$⊢\;X_{role\;}\left(pId\right)behavior\left(pId\right)\leftarrow \;O_{HDM\_BBCUser/HDM\_BBCProvider}BlockMaliciousContract$

ReplaceWithNonMaliciousMonitor&AssessmentContract

## 6. Theoretical Evaluation & Comparative Empirical Study Results and Discussion

This section discusses the results of the theoretical study based on the case study design methodology and the results of the empirical study. This section also discusses how the theoretical and evaluation results answer the study questions.

### 6.1. Theoretical Results

The theoretical results are presented as a collection of evidence in each phase of the proposed methodology through a theoretical evaluation study. The evidence that supports the study propositions is presented in Table 2. From the HDM case study, we identified ten security goals, eleven stakeholders, six policies, and four guard policy violation conditions in phase 1 (monitoring). In phase 2 (threat analysis), we identified 8 SC vulnerabilities; 22 invoked SLA SC; 4 reasoning concepts; 7 state concepts; 4 vulnerability concepts; V1, V2, V3, and V4 as vulnerability IDs; and SC1, SC2, SC3, SC4, SC5, SC6, and SC7 as state IDs. In phase 3 (planning), we identified four security countermeasures, four security requirements for mitigating the identified vulnerability, 6 SC agents, four behavior concepts, and six responsible agent concepts. Four security requirements were executed to mitigate the vulnerabilities identified in phase 4 (execution).

The evidence collected in each phase presented in Table 2 demonstrates how the four phases of the self-adaptive security RE_BBC conform to the study questions (SQ1 and SQ2) and validates the general propositions and their specific propositions claimed in Table 1.

### 6.2. Feasibility Study Result

This section discusses the results of the feasibility study. Table 4 presents the results of the feasibility study for the HDM case study based on the study questions (SQ3, SQ4, and SQ5), hypotheses, and parameters defined for the empirical study in Section 3.2. A statistical test is conducted on the empirical study of the HDM system, which includes mean, median, standard deviation, and *p*-value calculations. These values are validated using a statistical test based on test statistics of the unequal variance *t*-test [10] and Mann–Whitney *U* test [11] with a nominal α value of 0.05. Based on the *p*-values (*p*-value < 0.05), we can reject all null hypotheses defined in Table 3.

Regarding the healthcare case study, the statistical results indicate better performances for all parameters, namely, the quality of artifacts (%), evaluation quality (%), efficiency (min), complexity level, and usefulness level for our proposed self-adaptive security RE_BBC approach when compared to the SQUARE approach. The mean values for the quality of artifacts (%) is 48.54 for the SQUARE approach and 89.65 for the proposed approach. The mean value of the self-adaptive security evaluation quality (%) indicates a substantial difference. The values are 39.38 for the SQUARE approach and 87.49 for our proposed approach. Based on the efficiency with respect to the time duration for applying both approaches, there was a significant time difference of approximately one-third compared with the proposed approach.

Regarding rating complexity and usefulness of both approaches, SMEs used a four-level score that ranged between levels 1: very complex, 2: complex, 3: simple, and 4: very simple for complexity and levels 1: not useful, 2: less useful, 3: useful, and 4: very useful for usefulness. The complexity mean values are 1.66 and 3.66 for the SQUARE methodology and our proposed approach, respectively. The complexity mean value ratings indicate that our proposed approach is significantly simpler than the SQUARE approach. Similarly, the usability mean value of 1.66 and 3.60 for SQUARE methodology and the proposed approach, respectively, indicates that SMEs found our proposed approach useful in providing self-adaptive security for SLA-based SC for HDM BBC systems.

### 6.3. Comparative Analysis between the Proposed SRE_BBC Approach and SQUARE Approach Based on Feasibility Study Results

**Figure 12:**
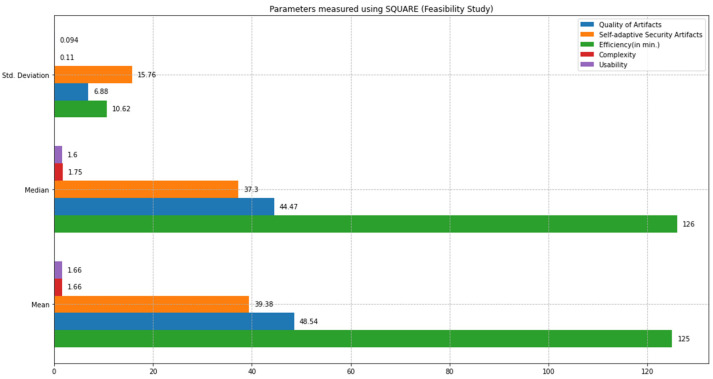
Statistical test for parameters measured using SQUARE approach for feasibility study.

**Figure 13:**
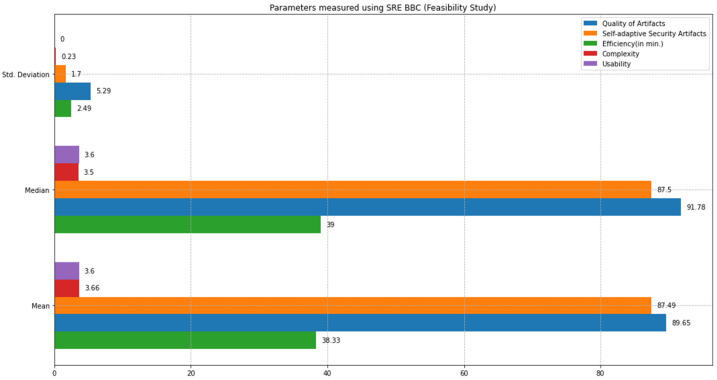
Statistical test for parameters measured using SRE_BBC approach for feasibility study.

This section compares the state-of-the-art SQUARE method and the proposed SRE_BBC approach based on the feasibility study results. We comparatively analyzed the performance of both approaches based on statistical tests, including mean, median, standard deviation, and *p*-values calculations using test statistics of the unequal variance *t*-test [10] and Mann–Whitney *U* test [11]. We defined parameters such as quality of artifacts, self-adaptive security evaluation quality, efficiency, complexity, and usability to compare both approaches. Figure 12 graphically represents the mean, median, and standard deviation bar graphs (in different colors) for each parameter, including quality of artifacts, self-adaptive security artifacts quality, efficiency, complexity, and usability measured using the SQUARE approach. Figure 13 graphically represents the mean, median, and standard deviation bar graphs using the proposed SRE_BBC approach. Figure 13 graph shows better performance for all parameters for our proposed approach when compared to the SQUARE approach in Figure 12.

The quality of artifacts parameter in the feasibility study is evaluated based on the dependent variables, including the number of security goals, number of stakeholders, and number of security requirements.

**Figure 14:**
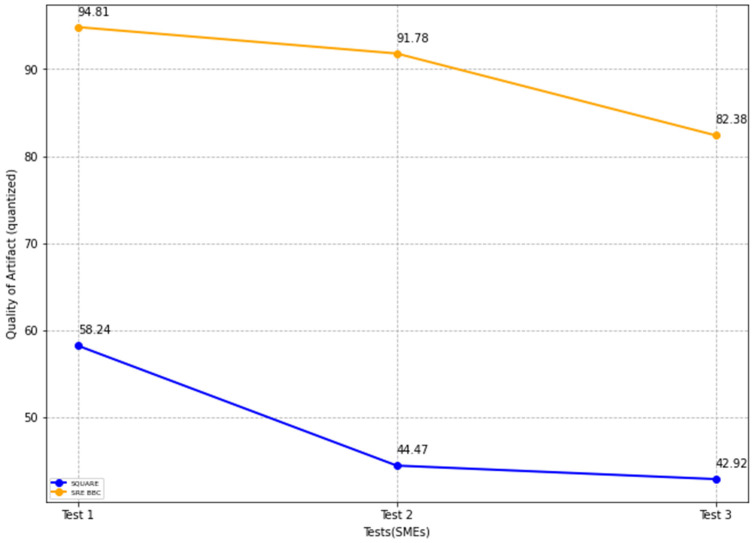
The comparison of quantified artifact quality for SQUARE and SRE_BBC (Feasibility Study).

The final quality of artifact values for the SQUARE approach and the proposed approach, as in Table 4, is calculated by averaging the values of the quality of artifact values of group 1 SMEs: SMEs 1, 2, and 3 for the SQUARE approach and group 2 SMEs: SME 4, 5, and 6 for the proposed SRE_BBC approach. In Figure 14, the blue and yellow lines show the average value of quality of artifacts for the SQUARE approach and the proposed SRE_BBC approach, respectively. As we can see in Figure 14, SMEs gave better performance for test 1 (58.24 vs. 94.81 mean), test 2 (44.47 vs. 91.78 mean), and test 3 (42.92 vs. 82.38 mean) when they used our proposed approach relative to SQUARE approach in terms of artifacts quality for HDM system.

The self-adaptive security evaluation quality parameter in the feasibility study is evaluated based on the dependent variables including the number of SC vulnerabilities identified, number of SLA SC invoked, number of security countermeasures provisioned, and number of SC agents assigned.

**Figure 15:**
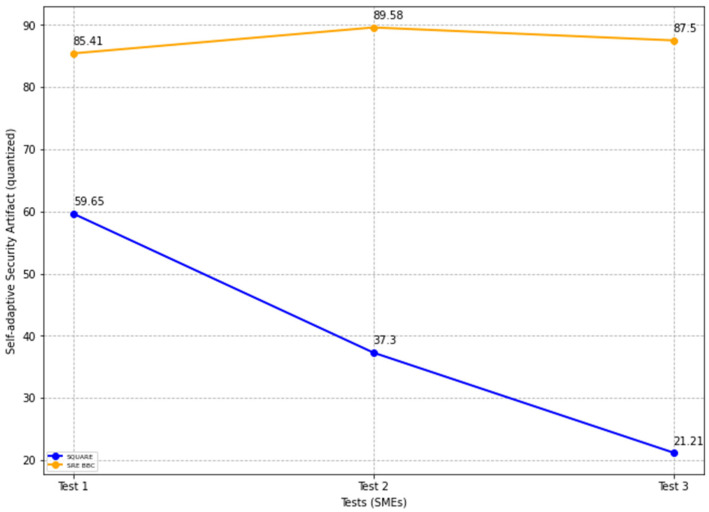
The comparison of quantified self-adaptive security evaluation quality for SQUARE and SRE_BBC (Feasibility Study).

The final self-adaptive security evaluation quality values for the SQUARE approach and the proposed approach, as in Table 4, are calculated by averaging the values of the self-adaptive security evaluation quality of group 1 SMEs: SMEs 1, 2, and 3 for the SQUARE approach and group 2 SMEs: SME 4, 5, and 6 for the proposed SRE_BBC approach. In Figure 15, the blue and yellow lines show the average value of self-adaptive security evaluation quality for the SQUARE approach and the proposed SRE_BBC approach, respectively. As we can see in Figure 15, SMEs gave a better performance for test 1 (59.65 vs. 85.41 mean), test 2 (37.3 vs. 89.58 mean), and test 3 (21.21 vs. 87.5 mean) for our proposed approach relative to the SQUARE approach in terms of self-adaptive security evaluation quality for HDM system.

We also calculated the complexity and usability parameters to assess the practicality of our proposed approach to providing self-adaptive security for BBC systems.

The complexity score is measured based on comprehensibility, simplicity, intuitiveness, and sensitivity parameters. Comprehensibility measures the quality of understandability of the method. Simplicity refers to the quality of a method being simple or uncompounded. Intuitive refers to the ability to understand a methodology without using rational processes. Finally, the sensitivity means that a method can respond to affective changes in the domain environment. As per the ratings, the simplicity and intuitiveness of our proposed approach were rated slightly low because the SMEs demanded more practice with the method. For example, the method’s threat analysis and planning phase required more training.

The final complexity level scores for the SQUARE approach and the proposed approach, as stated in Table 4, are calculated by averaging the complexity level scores of group 1 SMEs: SMEs 1, 2, and 3 for the SQUARE approach and group 2 SMEs: SME 4, 5, and 6 for the proposed SRE_BBC approach. SMEs used a four-level score for rating complexity that ranged between levels 1: very complex, 2: complex, 3: simple, and 4: very simple.

**Figure 16:**
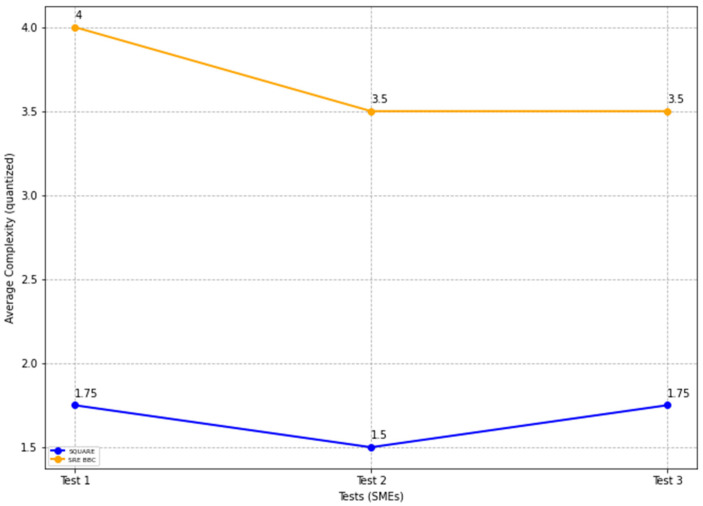
The comparison of quantified complexity level score for SQUARE and SRE_BBC (Feasibility Study).

In Figure 16, the blue and yellow lines show the comparison of the average complexity level score for the SQUARE approach and the proposed SRE_BBC approach. As we can see in Figure 16, SMEs gave better performance for test 1 (1.75 vs. 4 mean), test 2 (1.5 vs. 3.5 mean), and test 3 (1.75 vs. 3.5 mean) when they used our proposed approach relative to SQUARE approach in terms of complexity level score for HDM system.

The usability score is measured based on the average score of the related variables parameters based on the contribution of achieved self-adaptive security of SLA-based SC for HDM BBC systems. The parameters are the ability to elicit security goals, ability to elicit security requirements, ability to detect and analyze SC vulnerabilities, ability to protect and prevent using potential solutions, and methodology support and usefulness of the method.

**Figure 17:**
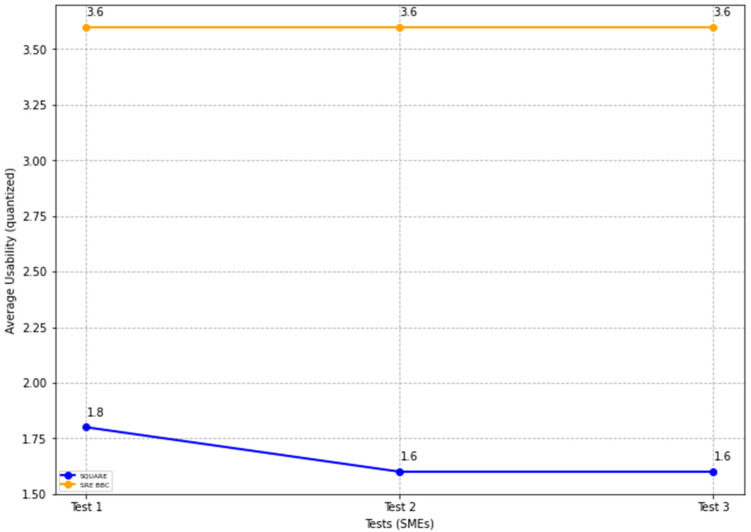
The comparison of quantified usability level score for SQUARE and SRE_BBC (Feasibility Study).

The final usability level scores for the SQUARE approach and the proposed approach, as stated in Table 4, are calculated by averaging the usability level scores of group 1 SMEs: SMEs 1, 2, and 3 for the SQUARE approach and group 2 SMEs: SME 4, 5, and 6 for the proposed SRE_BBC approach. SMEs used a four-level score for rating usability that ranged between levels 1: not useful, 2: less useful, 3: useful, and 4: very useful for usefulness. In Figure 17, the blue line and the yellow line show the average usability level score for the SQUARE approach and the proposed SRE_BBC approach, respectively. As we can see in Figure 17, SMEs gave better performance for test 1 (1.8 vs. 3.6 mean), test 2 (1.6 vs. 3.6 mean), and test 3 (1.6 vs. 3.6 mean) when they used our proposed approach relative to SQUARE approach in terms of usability level score for HDM system.

### 6.4. Replicated Study Result

This section discusses the results of the replicated studies. Table 5 presents the results of the replicated study for the HDM case study based on the study questions (SQ3, SQ4, and SQ5), hypothesis, and parameters defined for the empirical study in Section 3.2. To ensure consistency in data collection, the variables in the replicated study were converted in the same manner as in the feasibility study. Therefore, from the results in Table 5, we can see that the results from the replicated study are equivalent to the feasibility study results.

The results show that the SMEs with the proposed self-adaptive security RE_BBC outperformed the SMEs with the SQUARE approach in terms of artifact quality. The mean value of the quality of artifacts (%) was 53.55 for the SQUARE approach and 97.35 for the proposed approach. Similarly, the proposed approach performed better in terms of the quality of self-adaptive security evaluation. The mean value of the self-adaptive security evaluation quality (%) showed a substantial difference. The values are 51.79 for the SQUARE approach and 93.05 for the proposed approach. Compared with efficiency for the time duration for applying both approaches, the SQUARE approach required more time to perform the steps. The SMEs agree that the proposed approach is relatively easy to use as the complexity mean values are 1.75 and 3.83 for SQUARE and our proposed approach, respectively. The complexity mean value ratings show that our proposed approach tends to be extremely simple compared to the SQUARE approach. Similarly, the usability mean value of 1.53 and 3.60 for SQUARE and the proposed approach, respectively, shows that the SMEs found our proposed approach useful to address self-adaptive security for SLA-based SC for HDM BBC system.

### 6.5. Comparative Analysis between the Proposed SRE_BBC Approach and SQUARE Approach Based on Replicated Study Results

**Figure 18:**
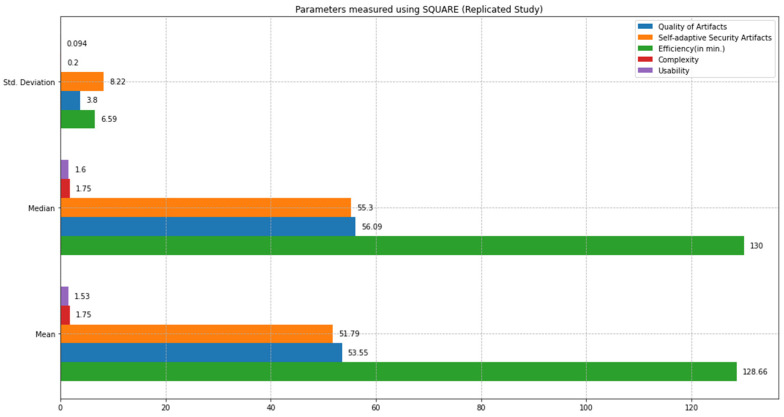
Statistical test for parameters measured using SQUARE approach for replicated study.

**Figure 19:**
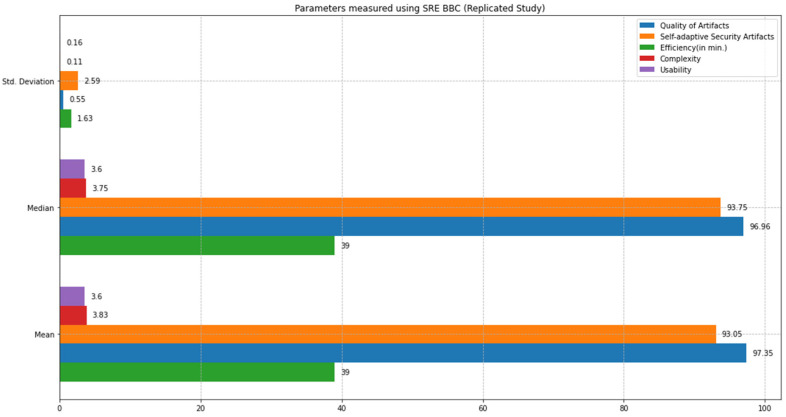
Statistical test for parameters measured using SRE_BBC approach for replicated study.

This section provides a comparative analysis of the state-of-the-art SQUARE method and the proposed SRE_BBC approach based on the replicated study results. Similar to the feasibility study, we comparatively analyzed the performance of both approaches based on statistical tests, including mean, median, standard deviation, and *p*-values calculations using test statistics of the unequal variance *t*-test [10] and Mann–Whitney *U* test [11]. Figure 18 graphically represents the mean, median, and standard deviation bar graphs (in different colors) for each parameter, including quality of artifacts, self-adaptive security artifacts quality, efficiency, complexity, and usability measured using the SQUARE approach. Figure 19 graphically represents the mean, median, and standard deviation bar graphs for each of these parameters using the proposed SRE_BBC approach. Figure 19 graph shows better performance for all parameters for our proposed approach when compared to the SQUARE approach in Figure 18.

Similar to the feasibility study, the average value of the quality of artifacts (%) in the replicated study was calculated based on the average of the percentage values of all artifacts, including the number of security goals, number of stakeholders, and number of security requirements.

**Figure 20:**
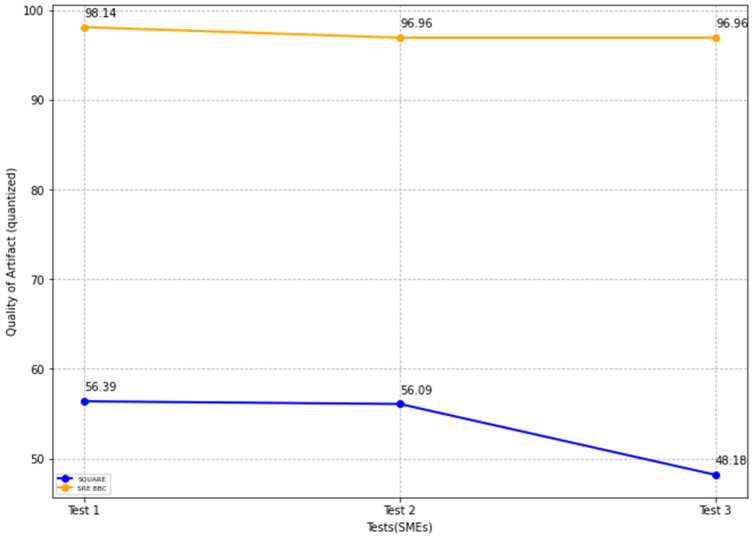
The comparison of quantified average artifact quality for SQUARE and SRE_BBC (Replicated Study).

The final value of the quality of artifacts for the SQUARE approach, stated in Table 5, is calculated by averaging the quality of artifact values of group 2 SMEs, SMEs 4, 5, and 6 for the SQUARE approach, and group 1 SMEs, SME 1, 2, and 3 for the proposed approach SRE_BBC. We discovered that SMEs in group 1 captured more security requirements and goals compared with the existing standards of the HDM systems. In Figure 20, the blue and yellow lines show the average value of quality of artifacts for the SQUARE approach and the proposed SRE_BBC approach, respectively. As we can see in Figure 20, SMEs gave better performance for test 1 (56.39 vs. 98.14 mean), test 2 (56.09 vs. 96.96 mean), and test 3 (48.18 vs. 96.96 mean) when they used our proposed approach relative to SQUARE approach in terms of artifacts quality for HDM system.

In the replicated study, we calculated the self-adaptive security evaluation quality parameter based on self-adaptive security artifacts, including the number of SC vulnerabilities identified, number of SLA SC invoked, number of security countermeasures provisioned, and number of SC agents assigned. As a result, the self-adaptive security evaluation quality was slightly higher than the feasibility study. Furthermore, we obtained a full score for the number of SLA SC invoked when SMEs used the proposed approach as SLA SC states were explained, and guidelines were given to the SMEs.

The final value of the self-adaptive security evaluation quality for the SQUARE approach and the proposed approach in Table 5 is calculated by averaging the self-adaptive security evaluation quality values of group 2 SMEs, SMEs 4, 5, and 6 for the SQUARE approach, and group 1 SMEs, SME 1, 2, and 3 for SRE_BBC approach.

**Figure 21:**
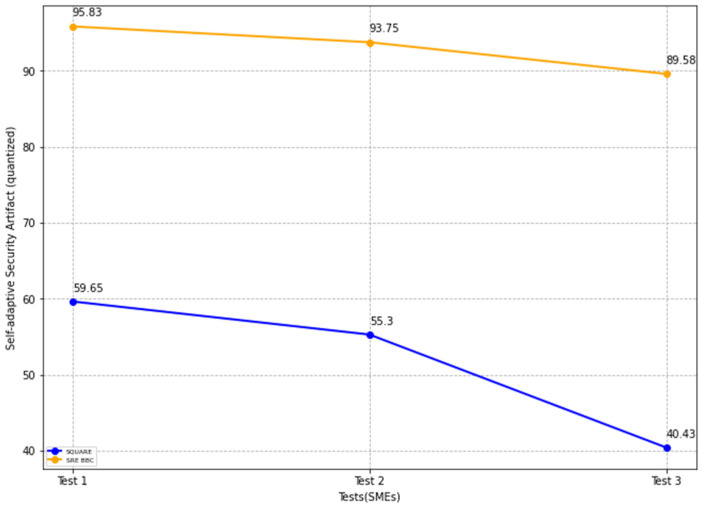
The comparison of quantified self-adaptive security evaluation quality for SQUARE and SRE_BBC (Replicated Study).

In Figure 21, the blue and yellow lines show the average value of self-adaptive security evaluation quality for the SQUARE approach and the proposed SRE_BBC approach, respectively. From Figure 21, SMEs gave better performance for test 1 (59.65 vs. 95.83 mean), test 2 (55.3 vs. 93.75 mean), and test 3 (40.43 vs. 89.58 mean) when they used our proposed approach relative to the SQUARE approach in terms of self-adaptive security evaluation quality for HDM system.

We also calculated the complexity and usability parameters to assess the practicality of our proposed approach to providing self-adaptive security for BBC systems. The complexity score is measured based on comprehensibility, simplicity, intuitiveness, and sensitivity parameters.

Similar to the feasibility study results, the replicated study results indicate that the proposed approach can address self-adaptive security more quickly than the SQUARE approach. The final complexity level score for the SQUARE approach, and the proposed approach, as stated in Table 5, are calculated by averaging the complexity level scores of group 2 SMEs and SMEs 4, 5, and 6 for the SQUARE approach and group 1 SMEs, SME 1, 2, and 3 for the proposed SRE_BBC approach. SMEs used a four-level score for rating complexity that ranged between levels 1: very complex, 2: complex, 3: simple, and 4: very simple.

**Figure 22:**
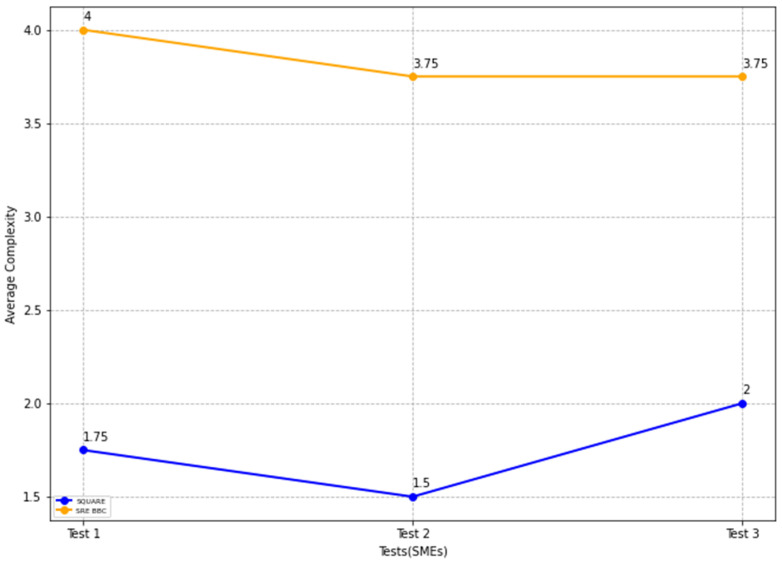
The comparison of quantified average complexity level score for SQUARE and SRE_BBC (Replicated Study).

In Figure 22, the blue and yellow lines compare the average complexity level score for the SQUARE approach and the proposed SRE_BBC approach. As we can see in Figure 22, SMEs gave better performance for test 1 (1.75 vs. 4 mean), test 2 (1.5 vs. 3.75 mean), and test 3 (2 vs. 3.75 mean) when they used our proposed approach relative to the SQUARE approach in terms of complexity level score for HDM system.

The usability score is measured based on the parameters, including the ability to elicit security goals, ability to elicit security requirements, ability to detect and analyze SC vulnerabilities, ability to protect and prevent the use of potential solutions, and methodology support and usefulness of the method. The results of the usability score report similar results to those of the feasibility study, where the usability parameters are rated high for the proposed SRE_BBC approach.

The final usability level scores for the SQUARE approach and the proposed approach, as stated in Table 5, are calculated by averaging the usability level scores of group 2 SMEs: SMEs 4, 5, and 6 for the SQUARE approach and group 1 SMEs: SME 1, 2, and 3 for the proposed SRE_BBC approach. SMEs used a four-level score for rating usability that ranged between levels 1: not useful, 2: less useful, 3: useful, and 4: very useful for usefulness.

**Figure 23:**
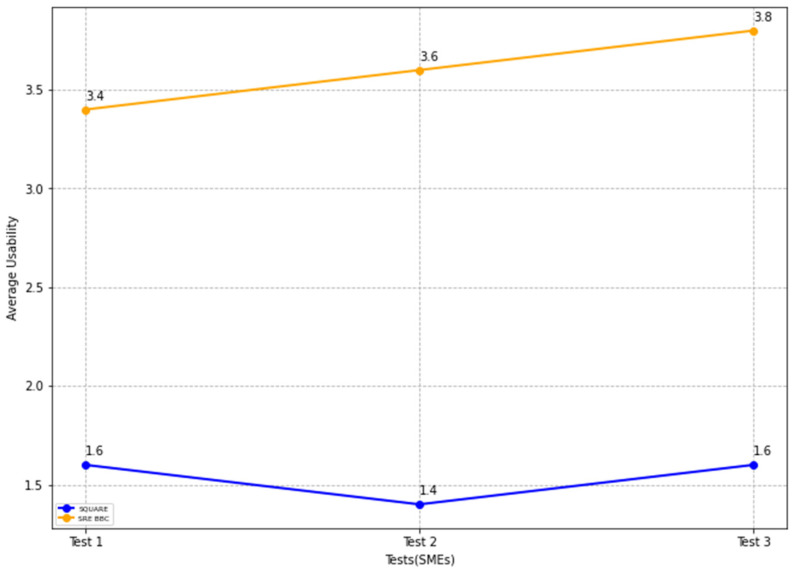
The comparison of quantified average usability level score for SQUARE and SRE_BBC (Replicated Study).

In Figure 23, the blue and yellow lines compare the average usability level score for the SQUARE approach and the proposed SRE_BBC approach. As we can see in Figure 23, SMEs gave better performance for test 1 (1.6 vs. 3.4 mean), test 2 (1.4 vs. 3.6 mean), and test 3 (1.6 vs. 3.8 mean) when they used our proposed approach relative to SQUARE approach in terms of usability level score for HDM system.

## 7. Threat to Validity

We followed the Systematic Literature Review (SLR) protocol [20] in conducting this study and analyzed 20 papers published from 2003 until 2021. We structured the search process both automatically and manually:

- We selected the search engines, including IEEE Xplore, Association for Computing Machinery (ACM) Digital Library, Springer Digital Library, Elsevier Science Direct, and Google Scholar.
- We searched these engines with definite keywords, including “self-adaptive security in Blockchain” and “self-adaptive security for smart contract”.
- We refined the search keywords as “self-adaptive security AND Blockchain,” “self-adaptive security AND smart contract,” and “self-adaptive security AND smart contract vulnerabilities”.
- We have attempted to minimize the threats to the validity of the findings in this paper.

Nevertheless, some possible threats need to be discussed. For example, there can be a possibility that some befitting papers were not identified by the search engine and therefore not included. We utilized numerous search engines to avoid this threat and included reputed and relevant journals and conferences. We have tried to balance broadening the search and getting feasible results.

## 8. Related Work

This section presents an overview of the state-of-the-art research related to self-adaptive security in RE, security requirements addressed using Blockchain, and self-adaptive security for the blockchain-based system. At the end of the related work, we also summarized how our work contributes and differentiates from the existing works.

### 8.1. Self-Adaptive Security in Requirements Engineering

Self-adaptive security is a research area studied and explored in requirements engineering in the last few years. In the early years, the works focused on reconfiguring the security policies at runtime [21] to maintain a separation of concerns between security policies and program specifications. There has been an increasing number of studies on requirements-aware self-adaptive security after 2008. Most of the works focus on following security requirements during design using goal modeling approaches. For example, Baresi et al. in [22] added self-adaptive goals to generalize KAOS (Knowledge Acquisition on Automated Specification) to embed adaptation countermeasures. Morandini et al. in [23] modified the requirements TROPOS goal model by introducing agents to model self-adaptive systems.

### 8.2. Security Requirements Addressed Using Blockchain

Blockchain has enabled the broader use of its features in supply chain management and cloud computing scenarios. Researchers have entrusted Blockchain to address different security requirements, such as data provenance in the cloud [24], trust issues among stakeholders [25], privacy [26,27], and integrity [28,29]. For example, in [24], a data provenance model for a cloud was proposed to maintain the integrity of honest mining operations in the blockchain cloud. In [25], the authors designed and implemented a blockchain to increase the trustworthiness of business process re-engineering. According to a bibliometric analysis [30], confidentiality and security management of data and information is the second most researched stream of available publications in Blockchain.

### 8.3. Self-Adaptive Security Using Smart Contracts

As a blockchain feature, the smart contract has enabled wider use in scenarios such as finance or data management. Most of the research on SC is conducted on the functionality of SC, and few researchers consider the security issues and security requirements for the complete life cycle of the contracts being used, right from the contract’s formation. Few studies have focused on providing partial self-adaptive security for SC; however, no studies have provided self-adaptive security for SLA-based SC vulnerabilities and attacks for BBC applications.

- Building Security Templates for Smart Contracts

Some studies concentrate on building security templates for SC. For example, Clark et al. [31,32] elicited critical requirements and design results for SC. However, they did not explicitly consider the security issues and security requirements for SC applications. Semantic modeling and rules exist for secure SC template formation [33]; however, existing methods for self-adaptive SC security still rely on human efforts for code writing and integration.

- Smart Contracts Used for Anomaly Detection

Smart contracts are used for anomaly detection in blockchain-based logs. For example, LSC [6] uses an SC to perform automatic online log analysis in blockchain-enabled log systems. Once an anomaly is discovered in the log, the security operator must demonstrate the type of anomaly. However, no techniques are used to support security operators in decision-making. In contrast, our approach provides threat models to support security operators/SC agents in threat analysis and decision-making. Another similar work is implementing security [34]. The authors used high-granularity metrics using function-based access control to detect malicious behavior in access control structures; however, they did not focus on self-adaptive mitigation strategies. However, our approach provides threat analysis and a security countermeasure solution for mitigating detected vulnerabilities. Some studies used tools to detect vulnerabilities in SC; for example, Luu et al. used the Oyente [1] tool to detect vulnerabilities in SC by removing the control-flow graph from the EVM bytecode of a contract. However, they do not provide self-adaptive security for mitigating security vulnerabilities.

- Building Formal Models for Monitoring Smart Contracts

Some studies have focused on improving security by building formal model clauses. For example, finite-state machines were used in [35] to build a secure design for SC. Guido et al. [11] formally represented countermeasure solutions as contract-to-duty obligations, using a BCL to mitigate violations of obligations in contracts. However, their approach lacks the self-adaptiveness to monitor contracts and provide countermeasure solutions at runtime. Some works are there where authors built their own SC language. For example, Shari et al. [36] built a formal specification language for legal contracts by using the legal concepts of the ontology. However, their language did not consider self-adaptive security concepts.

### 8.4. Contribution & Comparison of the Proposed Work with the State-of-the-Art Research

The critical contribution of the proposed approach was to develop a novel self-adaptive security RE_BBC process for SLA-based SC for BBC systems to detect security vulnerabilities and challenges and mitigate them by providing proactive counter-solutions. The proposed approach followed standard software lifecycle principles and a guided process to model secure and quality-enabled SC. In contrast, very few studies have focused on providing partial self-adaptive security for SC; however, no studies have provided self-adaptive security for SLA-based SC vulnerabilities and attacks for BBC applications. We also proposed the AS-BCL and AS-FCL formalisms and provided their mappings to the MAPE-BBC phases to provide self-adaptiveness. In contrast, the formal models for monitoring SC in these studies are not customized for self-adaptive security as they lack self-adaptive security concepts. We validated our research study with six subject matter experts who have 15+ years of experience in the software engineering field and are familiar with security concepts, Blockchain, SC, and cloud computing areas. We have statistically proved the research questions and hypotheses using the *t*-test [10] and Mann–Whitney *U* test [11]. In addition, we provided a comparative analysis between the state-of-the-art SQUARE method and the proposed SRE_BBC approach based on statistical tests including mean, median, standard deviation, and *p*-values calculations on the defined parameters such as quality of artifacts, self-adaptive security evaluation quality, efficiency, complexity, and usability. The validation results indicate that the proposed approach is more efficient and practical at providing self-adaptive security for SLA-based SC for BBC systems than the state-of-the-art SQUARE method.

## 9. Limitations and Recommendations for Future Research

Following are the limitation of the proposed approach and study. Due to the lack of a specialized secure SC testing tool for evaluating the formal models (AS-BCL and AS-FCL) used in this study, the SMEs had to manually map the concepts of formalism to the MAPE-BBC phases to evaluate captured adaptive security achievement. They evaluated the captured adaptive security achievement based on their defined security goals, requirements, SC vulnerabilities, security countermeasure solution, and SC agents. Another limitation found in the SLA-based SC assessment and validation phase was that some self-adaptive secure execution scenarios could not be tested in the health care domain because of the limited metrics and measurements.

The following questions are the current gaps and challenges inferred from the current research efforts for future research.

- How can we determine whether a given BBC system will be stable? How proactively can the BBC system respond to a change in the functional environment for respective applications? What are the hindrances that will affect the response time, and how to optimize them?
- How can we improve the accuracy of the self-adaptive security for BBC systems to predict the attack and achieve robust stability in different application domains? How much cost/time will the system spend in achieving accuracy and stability?

There are several aspects to improving self-adaptive security specialized tools to incorporate secure SC development and secure SC testing. However, with the help of a secure SC specialized tool, SC security engineers can create SC models using secure SC languages that can generate SC artifacts that can be maintained at runtime. Additionally, these models can be verified and validated to address self-adaptive security problems.

## 10. Conclusions

Building a BBC system with the capability to adapt itself and make proactive decisions when any threat is detected is not a trivial task. Threat models, goal models, SRE, and the MAPE-BBC process play a critical role in achieving self-adaptive security. This study aims to provide SC security developers with a self-adaptive security method to identify security threats and vulnerabilities in a BBC environment and take proactive solutions to mitigate these vulnerabilities. This study also examines how the proposed approach addresses self-adaptive security for securing SLA-based SC in the BBC and how the proposed approach is executed and evaluated to achieve it.

In this study, self-adaptive security RE_BBC was proposed to address several security vulnerabilities and attacks in an SLA-based SC for BBC systems. Self-adaptive security RE_BBC is a novel and promising framework for integrating self-adaptive security into the RE_BBC process. The proposed framework intends to enhance the SC security developer’s ability to determine security vulnerabilities and threats in the SC and provide security countermeasures as security requirements to execute and achieve self-adaptive security in BBC systems.

The proposed approach is applied to an HDM case study and validated theoretically using study propositions and collected evidence. The feasibility and replicated studies’ results demonstrate that the proposed approach effectively identifies the security goals, SC vulnerabilities, and gathers security requirements, and provides countermeasures to mitigate the vulnerabilities via the SC agents and address self-adaptive security in SC development for the BBC time-efficient manner. Moreover, the study demonstrates that the proposed approach is more realistic for addressing self-adaptive security in BBC systems. Furthermore, it could help SC security developers identify self-adaptive security artifacts, which would assist them in identifying SC security threats and vulnerabilities and provide security solutions to mitigate them to achieve self-adaptive security.

## Figures and Tables

**Table 1:** Case study propositions and units of analysis.

Study Questions	Study Propositions	Description	Units of Analysis
**SQ1**	SP1.1	The proposed approach detects vulnerabilities such as ToD, timestamp dependence, mishandled exceptions, and reentrancy vulnerability in the SLA-based SC transaction log and call log using phase monitoring and identifies these vulnerabilities using threat models in the threat analysis phase.	SLA security requirements, case study scenarios, threat models, goal models, AS-BCL and AS-FCL formulism.
**SQ1**	SP1.2	The proposed approach plans to provide countermeasures using the threat model, goal model, and SLA security specifications.	Threat models, goal models, SLA security specifications, and AS-BCL and AS-FCL formulism.
**SQ1**	SP1.3	The proposed approach detects any new types of vulnerabilities, which will be listed under the mishandled exception if any contract does not respond with its return value or if there is a malicious contract or user controlling the SC.	Threat model
**SQ2**	SP2.1	The proposed approach executes countermeasures to mitigate the vulnerability and achieve security goals via self-adaptive agents.	SLA security specifications, responsible self-adaptive agents.
**SQ2**	SP2.1	The proposed approach validates the security requirements by checking that the resulting SLA-based SC specifications are persistent, correct, absolute, asserted, and valid by monitoring and evaluating the specified self-adaptive security requirements using secure service execution scenarios.	Secure service execution scenarios
**SQ2**	SP2.3	The proposed approach evaluates the self-adaptive security concepts by mapping the AS-BCL and AS-FCL formalization concepts (guard, reasoning, vulnerability, behavior, and responsible agent) with the MAPE-BBC phases and verifies the values.	AS-BCL and AS-FCL formulism

**Table 2:** Evidence Collection through Vulnerability Identification, and Countermeasures Summary Result.

Questions (Units)	Evidence Captured by SMEs (Vulnerabilities Identification, and Countermeasures Summary Report)	Step/Phase/Method in Self- Adaptive Security RE_BBC Process	Related Propositions That Support/Reject
SQ1 (Monitor)	SMEs record the *security goal(s)* from the security requirements.	Phase 1 (Monitor)	General Proposition (GP1) Study Propositions (SP): SP1.1 SP1.3 SP2.3
SQ1	SMEs record the value of *policy* and *guard* concept from AS-BCL.		
SQ1	SMEs record the value of *name(pId)* and *guard* concept in the AS-FCL to check policy violation.		
SQ1 (Threat Analysis)	SMEs record the *smart contract vulnerabilities*, *invoked SLA smart contract* observed from each scenario of the case study.	Phase 2 (Threat Analysis)	GP1 SP1.1 SP1.3 SP2.3
SQ1	SMEs record the *smart contract vulnerabilities*, *use cases*, *invoked SLA states* from the goal model.		
SQ1	SMEs record the *type of vulnerability* from threat model for each scenario.		
SQ1	SMEs record the value of *reasoning*, *state*, and *vulnerability* concepts from the AS-BCL.		
SQ1	SMEs record the value of *reasoning ID*, *vulnerability ID* and *state ID* concepts from the AS-FCL.		
SQ1 (Plan)	SMEs record the *security countermeasures* from the goal model for each type of security vulnerability identified from the use cases.	Phase 3 (Planning)	GP1 SP1.2 SP2.3
SQ1	SMEs record the specific *security requirement* for the mitigating the identified vulnerability.		
SQ1	SMEs record the *smart contract agent*, responsible for providing the specific security countermeasures from the goal model as well as from the security specifications.		
SQ1	SMEs record the value of *behavior* concept from the AS-BCL.		
SQ1	SMEs record the value of $R_{agent\left(pId\right)}behavior\left(pId\right)$ concept from the AS-FCL.		
SQ2 (Execute)	SMEs record the specific *security requirement* to mitigate the vulnerability and validate using secure service execution scenarios.	Phase 4 (Execute)	GP2 SP2.1 SP2.3
SQ2	SMEs record the value of *policy* and *Smart Contract agent* concept from the AS-BCL.		
SQ2	SMEs record the value of *policy* and *Xrole(pId)behavior(pId)* concept from the AS-FCL.		

**Table 3:** Study Questions, Hypothesis and Parameters defined for Empirical Study.

Study Questions	Null Hypothesis	Parameters
SQ3: Does the proposed approach efficaciously identify the SC vulnerabilities, gather security requirements, and provide countermeasures to mitigate the vulnerabilities via the SC agents? (Quantitative)	There is no variation in terms of effectiveness of quality of artifacts gathered via the SQUARE and the proposed Self-Adaptive Security RE_BBC approach. ($H_{1.1_{0}}$)	No. of security goals, No. of stakeholders, No. of security requirements, and No. of operations/use cases.
	There is no variation in terms of self-adaptive security evaluation quality parameters collected with the proposed approach and SQUARE approach. ($H_{1.2_{0}}$)	No. of SC vulnerabilities identified, No. of SLA SC invoked, No. of security countermeasure identified, and No. of SC agents assigned.
SQ4: Does the proposed approach time-efficiently address self-adaptive security in the SC development for BBC? (Time efficiency)	There is no variation in terms of efficiency between addressing self-adaptive security with the proposed approach and with the SQUARE approach. ($H_{2.1_{0}}$)	Time duration (mins) calculated for each phase of the security process.
SQ5: Is the proposed approach considered to be realistic to guide the SC developer to address self-adaptive security for BBC systems in terms of usability and complexity level? (Qualitative)	There is no variation in terms of usability between addressing self-adaptive security with the proposed approach and the SQUARE approach. ($H_{3.1_{0}}$)	Ability to elicit security goals, ability to elicit security requirements, ability to detect & analyze SC vulnerabilities, ability to protect and prevent using potential solutions, and methodology support and usefulness. Average Usability (Level→1: Very less useful, 2: less useful, 3: useful, 4: very useful)
	There is no variation in terms of complexity between addressing self-adaptive security with the self-adaptive security RE_BBC and without the proposed approach. ($H_{3.2_{0}}$)	Comprehensibility, simplicity, intuitiveness, sensibility/reasonability. Average Complexity (Level→1: Very complex, 2: complex, 3: simple, 4: very simple)

**Table 4:** Statistical test results on the feasibility study for healthcare data management system.

Parameters	SQUARE Approach			Self-Adaptive Security RE_BBC Approach (Proposed)			Statistical Test	*p*-Value
	Mean	Median	Std. Deviation	Mean	Median	Std. Deviation		
**Quality of Artifacts** **(%)**	48.54	44.47	6.88	89.65	91.78	5.29	*t*	0.00157490
**Self-adaptive Security Evaluation Quality** **(%)**	39.38	37.30	15.76	87.49	87.5	1.70	*t*	0.0326880
**Efficiency** **(mins)**	125	126	10.62	38.33	39	2.49	*t*	0.00342108
**Complexity** **(Level** **→1: Very Complex, 2: Complex, 3: Simple, 4: Very simple)**	1.66	1.75	0.11	3.66	3.50	0.23	U	0.02296
**Usefulness** **(Level** **→ 1: Very less useful, 2: Less useful, 3: Useful, 4: Very Useful)**	1.66	1.60	0.094	3.60	3.60	0	U	0.009321333

**Table 5:** “Statistical Test Results” based on the replicated study for healthcare data management system.

Parameters	SQUARE Approach			Self-Adaptive Security RE_BBC Approach (Proposed)			Statistical Test	*p*-Value
	Mean	Median	Std. Deviation	Mean	Median	Std. Deviation		
**Quality of Artifacts** **(%)**	53.55	56.09	3.80	97.35	96.96	0.55	*t*	0.00210090
**Self-adaptive Security Evaluation Quality (%)**	51.79	55.3	8.22	93.05	93.75	2.59	*t*	0.00811662
**Efficiency** **(mins)**	128.66	130	6.59	39	39	1.63	*t*	0.00105050
**Complexity** **(Level** **→1: Very Complex, 2: Complex, 3: Simple, 4: Very simple)**	1.75	1.75	0.20	3.83	3.75	0.11	U	0.0261166
**Usefulness** **(Level** **→ 1: Very less useful, 2: Less useful, 3: Useful, 4: Very Useful)**	1.53	1.6	0.094	3.60	3.60	0.16	U	0.009321333

## Data Availability

The data presented in this study are available in article or Supplementary Materials.

## Supplementary Materials

Supplementary Materials can be downloaded at: https://www.mdpi.com/article/10.3390/s22103903/s1.
