# Supplementary material for: SRE_BBC: A Self-Adaptive Security Enabled Requirements Engineering Approach for SLA Smart Contracts in Blockchain-Based Cloud Systems
Source: Sensors (Basel). 2022 May 21;22(10):3903. doi: 10.3390/s22103903 (PMC9143589; doi:10.3390/s22103903)
Supplement: Supplementary file 1 [file sensors-22-03903-s001.zip › sensors-1704551-supplementary.pdf]

Supplementary Material

Feasibility Study Tables

The average value of the quality of artifacts (%) was calculated based on the average of the percentage values of all artifacts, including no. of security goals, no. of stakeholders and no. of the security requirements as shown in Tables S1–S3.

The final quality of artifact values for the SQUARE approach, shown in Table 4 (main manuscript), are calculated by averaging the values of the quality of artifact values of group 1 SMEs: SME 1, 2, and 3 stated in Tables S1, S2 and S3, respectively. Similarly, to calculate the final quality of artifact values for our proposed approach, we took the average quality of artifact values of group 2 SMEs: SME 4, 5, and 6 in Tables S1, S2 and S3, respectively.

Table S1: "Quality of Artifacts" results obtained from the feasibility study conducted with randomly chosen SMEs

Artifacts	Case Study: Healthcare Data Management System	
	**SQUARE Approach Group 1 [SME 1]**	**Self-Adaptive Security RE_BBC Approach Group 2 [SME 4]**
No. of security goals	5 out of 10=50	9 out of 10=90
No. of stakeholders	7 out of 11=63.63	11 out of 11=100
No. of security requirements	11 out of 18=61.11	17 out of 18=94.44
**Average value of Quality of Artifacts (%)**	**58.24**	**94.81**

Table S2: "Quality of Artifacts" results obtained from the feasibility study conducted with randomly chosen SMEs

Artifacts	Case Study: Healthcare Data Management System	
	**SQUARE Approach Group 1 [SME 2]**	**Self-Adaptive Security RE_BBC Approach Group 2 [SME 5]**
No. of security goals	4 out of 10=40	9 out of 10=90
No. of stakeholders	6 out of 11=54.54	10 out of 11=90.90
No. of security requirements	7 out of 18=38.88	17 out of 18=94.44
**Average value of Quality of Artifacts (%)**	**44.47**	**91.78**

Table S3: "Quality of Artifacts" results obtained from the feasibility study conducted with randomly chosen SMEs

**Artifacts**	**Case Study: Healthcare Data Management System**	
	**SQUARE Approach Group 1 [SME 3]**	**Self-Adaptive Security RE_BBC Approach Group 2 [SME 6]**
No. of security goals	5 out of 10=50	8 out of 10=80
No. of stakeholders	5 out of 11=45.45	8 out of 11=72.72
No. of security requirements	6 out of 18=33.33	17 out of 18=94.44
**Average value of Quality of Artifacts (%)**	**42.92**	**82.38**

We also calculated the self-adaptive security evaluation quality by averaging the self-adaptive security artifacts, namely, the number of SC vulnerabilities identified, number of SLA SCs invoked, number of security countermeasures provisioned, and number of SC agents assigned shown in Tables S4–S6. We obtained a full score for the number of SLA SCs invoked when SMEs used the proposed approach as SLA SC states were explained and guidelines were provided to the SMEs.

The final value of the self-adaptive security evaluation quality for the SQUARE approach, stated in Table 4 (main manuscript), is calculated by averaging the self-adaptive security evaluation quality values of group 1 SMEs: SME 1, 2, and 3 presented in Tables S4, S5 and S6, respectively. Similarly, to calculate the final value of the self-adaptive security evaluation quality for the proposed approach, we averaged the self-adaptive security evaluation quality values of group 2 SMEs: SME 4, 5, and 6 presented in Tables S4, S5 and S6, respectively.

Table S4: "Self-adaptive Security Evaluation Quality Results" obtained from the feasibility study conducted with randomly chosen SMEs

**Self-adaptive security artifacts**	**Case Study: Healthcare Data Management System**	
	**SQUARE Approach Group 1 [SME 1]**	**Self-Adaptive Security RE_BBC Approach Group 2 [SME 4]**
No. of SC vulnerabilities identified	5 out of 8=62.50	7 out of 8=87.50
No. of SLA SC invoked	14 out of 22=63.63	22 out of 22=100
No. of security countermeasure provisioned	5 out of 8=62.50	7 out of 8=87.50
No. of SC agents assigned	3 out of 6=50	4 out of 6=66.66
**Average Self-adaptive Security Evaluation Quality (%)**	**59.65**	**85.41**

Table S5: Average "Self-adaptive Security Evaluation Quality Results" obtained from the feasibility study conducted with randomly chosen SMEs

**Self-adaptive security artifacts**	**Case Study: Healthcare Data Management System**	
	**SQUARE Approach Group 1 [SME 2]**	**Self-Adaptive Security RE_BBC Approach Group 2 [SME 5]**
No. of SC vulnerabilities identified	3 out of 8=37.5	7 out of 8=87.50
No. of SLA SC invoked	9 out of 22=40.90	22 out of 22=100
No. of security countermeasure identified	3 out of 8=37.5	7 out of 8=87.50
No. of SC agents assigned	2 out of 6=33.33	5 out of 6=83.33
**Average Self-adaptive Security Evaluation Quality (%)**	**37.30**	**89.58**

Table S6: Average "Self-adaptive Security Evaluation Quality Results" obtained from the feasibility study conducted with randomly chosen SMEs

**Self-adaptive security artifacts**	**Case Study: Healthcare Data Management System**	
	**SQUARE Approach Group 1 [SME 3]**	**Self-Adaptive Security RE_BBC Approach Group 2 [SME 6]**
No. of SC vulnerabilities identified	2 out of 8=25	6 out of 8=75
No. of SLA SC invoked	4 out of 22=18.18	22 out of 22=100
No. of security countermeasure identified	2 out of 8=25	6 out of 8=75
No. of SC agents assigned	1 out of 6=16.66	6 out of 6=100
**Average Self-adaptive Security Evaluation Quality (%)**	**21.21**	**87.5**

We also calculated the complexity and usability parameters to assess the practicality of our proposed approach in providing self-adaptive security for BBC systems. Tables S7–S9 report the complexity results, while Tables S10–S12 report the usability results.

Table S7: "Complexity Results" obtained from the feasibility study conducted with randomly chosen SMEs

Parameters	Case Study: Healthcare Data Management System	
	**SQUARE Approach Group 1 [SME 1]**	**Self-Adaptive Security RE_BBC Approach Group 2 [SME 4]**
Comprehensibility (Level→1: Very low, 2: low, 3: medium, 4: high)	2	4
Simplicity (Level→1: Very low, 2: low, 3: medium, 4: high)	2	4
Intuitive (Level→1: Very low, 2: low, 3: medium, 4: high)	1	4
Sensible/Reasonable (Level→1: Very low, 2: low, 3: medium, 4: high)	2	4
**Average Complexity (Level→1: Very Complex, 2: Complex, 3: Simple, 4: Very simple)**	**1.75**	**4**

Table S8: "Complexity Results" obtained from the feasibility study conducted with randomly chosen SMEs

Parameters	Case Study: Healthcare Data Management System	
	**SQUARE Approach Group 1 [SME 2]**	**Self-Adaptive Security RE_BBC Approach Group 2 [SME 5]**
Comprehensibility (Level→1: Very low, 2: low, 3: medium, 4: high)	1	3
Simplicity (Level→1: Very low, 2: low, 3: medium, 4: high)	2	4
Intuitive (Level→1: Very low, 2: low, 3: medium, 4: high)	2	4
Sensible/Reasonable	1	3

(Level→1: Very low, 2: low, 3: medium, 4: high)		
**Average Complexity (Level→1: Very Complex, 2: Complex, 3: Simple, 4: Very simple)**	**1.5**	**3.5**

Table S9: "Complexity Results" obtained from the feasibility study conducted with randomly chosen SMEs

**Parameters**	**Case Study: Healthcare Data Management System**	
	**SQUARE Approach Group 1 [SME 3]**	**Self-Adaptive Security RE_BBC Approach Group 2 [SME 6]**
Comprehensibility (Level→1: Very low, 2: low, 3: medium, 4: high)	2	4
Simplicity (Level→1: Very low, 2: low, 3: medium, 4: high)	2	3
Intuitive (Level→1: Very low, 2: low, 3: medium, 4: high)	1	3
Sensible/Reasonable (Level→1: Very low, 2: low, 3: medium, 4: high)	2	4
**Average Complexity (Level→1: Very Complex, 2: Complex, 3: Simple, 4: Very simple)**	**1.75**	**3.5**

Tables S10–S12 demonstrate that the usability parameters were rated highly for the self-adaptive security RE_BBC approach.

The final usability level score for the SQUARE approach, stated in Table 4 (main manuscript), is calculated by averaging the usability level scores of group 1 SMEs: SME 1, 2, and 3 stated in Tables S10, S11 and S12, respectively. Similarly, to calculate the final usability level for the proposed approach, stated in Table 4 (main manuscript), we averaged the usability level scores of group 2 SMEs: SME 4, 5, and 6 stated in Tables S10, S11 and S12, respectively.

Table S10: "Usability Results" obtained from the feasibility study conducted with randomly chosen SMEs

`	**Case Study: Healthcare Data Management System**	
	**SQUARE Approach Group 1[SME 1]**	**Self-Adaptive Security RE_BBC Approach Group 2 [SME 4]**
Ability to elicit security goals (Level→1: Very low, 2: low, 3: medium, 4: high)	2	4
Ability to elicit security requirements (Level→1: Very low, 2: low, 3: medium, 4: high)	2	3
Ability to detect & analyze SC vulnerabilities (Level→1: Very low, 2: low, 3: medium, 4: high)	1	4
Ability to protect and prevent using potential solutions (Level→1: Very low, 2: low, 3: medium, 4: high)	2	4
Methodology support and usefulness (Level→1: Very low, 2: low, 3: medium, 4: high)	2	3
**Average Usability (Level→1: Very less useful, 2: less useful, 3: useful, 4: Very useful)**	**1.8**	**3.6**

Table S11: "Usability Results" obtained from the feasibility study conducted with randomly chosen SMEs

**Parameters**	**Case Study: Healthcare Data Management System**	
	**SQUARE Approach Group 1[SME 2]**	**Self-Adaptive Security RE_BBC Approach Group 2 [SME 5]**
Ability to elicit security goals (Level→1: Very low, 2: low, 3: medium, 4: high)	1	4
Ability to elicit security requirements (Level→1: Very low, 2: low, 3: medium, 4: high)	2	4

Ability to detect & analyze SC vulnerabilities (Level→1: Very low, 2: low, 3: medium, 4: high)	2	3
Ability to protect and prevent using potential solutions (Level→1: Very low, 2: low, 3: medium, 4: high)	1	3
Methodology support and usefulness (Level→1: Very low, 2: low, 3: medium, 4: high)	2	4
**Average Usability (Level→1: Very less useful, 2: less useful, 3: useful, 4: Very useful)**	**1.6**	**3.6**

Table S12: "Usability Results" obtained from the feasibility study conducted with randomly chosen SMEs

**Parameters**	**Case Study: Healthcare Data Management System**	
	**SQUARE Approach Group 1 [SME 3]**	**Self-Adaptive Security RE_BBC Approach Group 2 [SME 6]**
Ability to elicit security goals (Level→1: Very low, 2: low, 3: medium, 4: high)	2	4
Ability to elicit security requirements (Level→1: Very low, 2: low, 3: medium, 4: high)	2	3
Ability to detect & analyze SC vulnerabilities (Level→1: Very low, 2: low, 3: medium, 4: high)	1	4
Ability to protect and prevent using potential solutions (Level→1: Very low, 2: low, 3: medium, 4: high)	1	3
Methodology support and usefulness (Level→1: Very low, 2: low, 3: medium, 4: high)	2	4
**Average Usability (Level→1: Very less useful, 2: less useful, 3: useful, 4: Very useful)**	**1.6**	**3.6**

Replicated Study Tables

The final value of the quality of artifacts for the SQUARE approach, stated in Table 5 (main manuscript), is calculated by averaging the quality of artifact values of group 2 SMEs, SME 4, 5, and 6 stated in Tables S13, S14 and S15, respectively. Similarly, to calculate the final quality of artifacts values for our proposed approach, we averaged quality of artifact values of group 1 SMEs, SME 1, 2, and 3 stated in Tables S13, S14 and S15, respectively. We detected that SMEs in group 1 captured more security requirements and goals compared with the existing standards of the healthcare data management systems.

Table S13: "Quality of Artifacts Results" obtained from the replicated study conducted with randomly chosen SMEs

**Artifacts**	**Case Study: Healthcare Data Management System**	
	**SQUARE Approach Group 2 [SME 4]**	**Self-Adaptive Security RE_BBC Approach Group 1 [SME 1]**
No. of security goals	5 out of 10=50	10 out of 10=100
No. of stakeholders	7 out of 11=63.63	11 out of 11=100
No. of security requirements	10 out of 18=55.55	17 out of 18=94.44
**Average value of Quality of Artifacts (%)**	**56.39**	**98.14**

Table S14: "Quality of Artifacts Results" obtained from the replicated study conducted with randomly chosen SMEs

**Artifacts**	**Case Study: Healthcare Data Management System**	
	**SQUARE Approach Group 2 [SME 5]**	**Self-Adaptive Security RE_BBC Approach Group 1 [SME 2]**
No. of security goals	4 out of 10=40	10 out of 10=100
No. of stakeholders	8 out of 11=72.72	10 out of 11=90.90
No. of security requirements	10 out of 18=55.55	18 out of 18=100
**Average value of Quality of Artifacts (%)**	**56.09**	**96.96**

Table S15: "Quality of Artifacts Results" obtained from the replicated study conducted with randomly chosen SMEs

Artifacts	Case Study: Healthcare Data Management System	
	SQUARE Approach Group 2 [SME 6]	Self-Adaptive Security RE_BBC Approach Group 1 [SME 3]
No. of security goals	4 out of 10=40	10 out of 10=100
No. of stakeholders	6 out of 11=54.54	10 out of 11=90.90
No. of security requirements	9 out of 18=50	18 out of 18=100
**Average value of Quality of Artifacts (%)**	**48.18**	**96.96**

We calculated the self-adaptive security evaluation quality by averaging the self-adaptive security artifacts, such as the number of SC vulnerabilities identified, number of SLA SCs invoked, number of security countermeasures provisioned, and number of SC agents assigned as shown in Tables S16–S18. The result of the self-adaptive security evaluation quality was slightly higher than that of the feasibility study. We obtained a full score for the number of SLA SCs invoked when SMEs used the proposed approach as SLA SC states were explained, and guidelines were given to the SMEs.

The final value of the self-adaptive security evaluation quality for the SQUARE approach in Table 5 (main manuscript) is calculated by averaging the self-adaptive security evaluation quality values of group 2 SMEs, SME 4, 5, and 6 stated in Tables S16, S17 and S18, respectively. Similarly, to calculate the final self-adaptive security evaluation quality value for the proposed approach, we averaged the self-adaptive security evaluation quality values of group 1 SMEs, SME 1, 2, and 3 stated in Tables S16, S17 and S18, respectively.

Table S16: "Self-adaptive Security Evaluation Quality Results" obtained from the replicated study conducted with randomly chosen SMEs

Self-adaptive security artifacts	Case Study: Healthcare Data Management System	
	SQUARE Approach Group 2 [SME 4]	Self-Adaptive Security RE_BBC Approach Group 1 [SME 1]
No. of SC vulnerabilities identified	5 out of 8=62.50	8 out of 8=100
No. of SLA SC invoked	14 out of 22=63.63	22 out of 22=100
No. of security countermeasure provisioned	5 out of 8=62.50	8 out of 8=100

No. of SC agents assigned	3 out of 6=50	5 out of 6=83.33
**Average Self-adaptive Security Evaluation Quality (%)**	**59.65**	**95.83**

Table S17: "Average Self-adaptive Security Evaluation Quality Results" obtained from the replicated study conducted with randomly chosen SMEs

**Self-adaptive security artifacts**	**Case Study: Healthcare Data Management System**	
	**SQUARE Approach Group 2 [SME 5]**	**Self-Adaptive Security RE_BBC Approach Group 1 [SME 2]**
No. of SC vulnerabilities identified	4 out of 8=50	7 out of 8=87.50
No. of SLA SC invoked	12 out of 22=54.54	22 out of 22=100
No. of security countermeasure identified	4 out of 8=50	7 out of 8=87.50
No. of SC agents assigned	4 out of 6=66.66	6 out of 6=100
**Average Self-adaptive Security Evaluation Quality (%)**	**55.3**	**93.75**

Table S18: "Average Self-adaptive Security Evaluation Quality Results" obtained from the replicated study conducted with randomly chosen SMEs

**Self-adaptive security artifacts**	**Case Study: Healthcare Data Management System**	
	**SQUARE Approach Group 2 [SME 6]**	**Self-Adaptive Security RE_BBC Approach Group 1 [SME 3]**
No. of SC vulnerabilities identified	4 out of 8=50	7 out of 8=87.50
No. of SLA SC invoked	9 out of 22=40.90	22 out of 22=100
No. of security countermeasure identified	3 out of 8=37.5	7 out of 8=87.50
No. of SC agents assigned	2 out of 6=33.33	5 out of 6=83.33
**Average Self-adaptive Security Evaluation Quality (%)**	**40.43**	**89.58**

We also calculated the complexity and usability parameters to assess the practicality of our proposed approach in providing self-adaptive security for BBC systems. Tables S19–S21 report the complexity results, while Tables S22–S24 report the usability results. Similar to the results of the feasibility study, these results indicate that the proposed approach can address self-adaptive security more easily than the SQUARE approach.

The final complexity level score for the SQUARE approach, stated in Table 5 (main manuscript), is calculated by averaging the complexity level scores of group 2 SMEs and SME 4, 5, and 6 stated in Tables S19, S20 and S21, respectively. Similarly, to calculate the final complexity level score for our proposed approach, stated in Table 5 (main manuscript), we averaged the complexity level scores of group 1 SMEs, SME 1, 2, and 3 stated in Tables S19, S20 and S21, respectively.

Table S19: "Complexity Results" obtained from the replicated study conducted with randomly chosen SMEs

Parameters	Case Study: Healthcare Data Management System	
	SQUARE Approach Group 2 [SME 4]	Self-Adaptive Security RE_BBC Approach Group 1 [SME 1]
Comprehensibility (Level→1: Very low, 2: low, 3: medium, 4: high)	1	4
Simplicity (Level→1: Very low, 2: low, 3: medium, 4: high)	2	4
Intuitive (Level→1: Very low, 2: low, 3: medium, 4: high)	2	4
Sensible/Reasonable (Level→1: Very low, 2: low, 3: medium, 4: high)	2	4
**Average Complexity (Level→1: Very Complex, 2: Complex, 3: Simple, 4: Very simple)**	**1.75**	**4**

Table S20: "Complexity Results" from the replicated study conducted with randomly chosen SMEs

Parameters	Case Study: Healthcare Data Management System	
	SQUARE Approach Group 2 [SME 5]	Self-Adaptive Security RE_BBC Approach Group 1 [SME 2]
Comprehensibility (Level→1: Very low, 2: low, 3: medium, 4: high)	2	3

Simplicity (Level→1: Very low, 2: low, 3: medium, 4: high)	1	4
Intuitive (Level→1: Very low, 2: low, 3: medium, 4: high)	2	4
Sensible/Reasonable (Level→1: Very low, 2: low, 3: medium, 4: high)	1	4
**Average Complexity (Level→1: Very Complex, 2: Complex, 3: Simple, 4: Very simple)**	**1.5**	**3.75**

Table S21: "Complexity Results" obtained from the replicated study conducted with randomly chosen SMEs

**Parameters**	**Case Study: Healthcare Data Management System**	
	**SQUARE Approach Group 2 [SME 6]**	**Self-Adaptive Security RE_BBC Approach Group 1 [SME 3]**
Comprehensibility (Level→1: Very low, 2: low, 3: medium, 4: high)	3	4
Simplicity (Level→1: Very low, 2: low, 3: medium, 4: high)	2	4
Intuitive (Level→1: Very low, 2: low, 3: medium, 4: high)	2	3
Sensible/Reasonable (Level→1: Very low, 2: low, 3: medium, 4: high)	1	4
**Average Complexity (Level→1: Very Complex, 2: Complex, 3: Simple, 4: Very simple)**	**2**	**3.75**

Tables S22–S24 report similar results to those of the feasibility study, where the usability parameters are rated high for the self-adaptive security RE_BBC approach.

The final usability level score for the SQUARE approach, stated in Table 5 (main manuscript), is calculated by averaging the usability level scores of group 2 SMEs and SME 4, 5, and 6 stated in Tables S22, S23 and S24, respectively. Similarly, to calculate the final usability level score for the proposed approach, stated in Table 5 (main manuscript), we averaged usability scores of group 1 SMEs and SME 1, 2, and 3 stated in Tables S22, S23 and S24, respectively.

Table S22: "Usability Results" obtained from the replicated study conducted with randomly chosen SMEs

**Parameters**	**Case Study: Healthcare Data Management System**	
	**SQUARE Approach Group 2 [SME 4]**	**Self-Adaptive Security RE_BBC Approach Group 1 [SME 1]**
Ability to elicit security goals (Level→1: Very low, 2: low, 3: medium, 4: high)	2	3
Ability to elicit security requirements (Level→1: Very low, 2: low, 3: medium, 4: high)	2	3
Ability to detect & analyze SC vulnerabilities (Level→1: Very low, 2: low, 3: medium, 4: high)	1	4
Ability to protect and prevent using potential solutions (Level→1: Very low, 2: low, 3: medium, 4: high)	1	3
Methodology support and usefulness (Level→1: Very low, 2: low, 3: medium, 4: high)	2	4
**Average Usability (Level→1: Very less useful, 2: less useful, 3: useful, 4: Very useful)**	**1.6**	**3.4**

Table S23: "Usability Results" obtained from the replicated study conducted with randomly chosen SMEs

**Parameters**	**Case Study: Healthcare Data Management System**	
	SQUARE Approach Group 2 [SME 5]	Self-Adaptive Security RE_BBC Approach Group 1 [SME 2]
Ability to elicit security goals (Level→1: Very low, 2: low, 3: medium, 4: high)	2	3
Ability to elicit security requirements (Level→1: Very low, 2: low, 3: medium, 4: high)	1	4

Ability to detect & analyze SC vulnerabilities (Level→1: Very low, 2: low, 3: medium, 4: high)	1	4
Ability to protect and prevent using potential solutions (Level→1: Very low, 2: low, 3: medium, 4: high)	1	3
Methodology support and usefulness (Level→1: Very low, 2: low, 3: medium, 4: high)	2	4
**Average Usability (Level→1: Very less useful, 2: less useful, 3: useful, 4: Very useful)**	**1.4**	**3.6**

Table S24: "Usability Results" obtained from the replicated study conducted with randomly chosen SMEs

**Parameters**	**Case Study: Healthcare Data Management System**	
	SQUARE Approach Group 2 [SME 6]	Self-Adaptive Security RE_BBC Approach Group 1 [SME 3]
Ability to elicit security goals (Level→1: Very low, 2: low, 3: medium, 4: high)	**2**	**4**
Ability to elicit security requirements (Level→1: Very low, 2: low, 3: medium, 4: high)	**2**	**4**
Ability to detect & analyze SC vulnerabilities (Level→1: Very low, 2: low, 3: medium, 4: high)	**1**	**3**
Ability to protect and prevent using potential solutions (Level→1: Very low, 2: low, 3: medium, 4: high)	**1**	**4**
Methodology support and usefulness (Level→1: Very low, 2: low, 3: medium, 4: high)	**2**	**4**
**Average Usability (Level→1: Very less useful, 2: less useful, 3: useful, 4: Very useful)**	**1.6**	**3.8**
